# Reversible, Selective, Ultrawide‐Range Variable Stiffness Control by Spatial Micro‐Water Molecule Manipulation

**DOI:** 10.1002/advs.202102536

**Published:** 2021-08-27

**Authors:** Inho Ha, Minwoo Kim, Kyun Kyu Kim, Sukjoon Hong, Hyunmin Cho, Jinhyeong Kwon, Seonggeun Han, Yeosang Yoon, Phillip Won, Seung Hwan Ko

**Affiliations:** ^1^ Soft Robotics Research Center Seoul National University 1 Gwanak‐ro, Gwanak‐gu Seoul 08826 Korea; ^2^ Applied Nano and Thermal Science Lab Department of Mechanical Engineering Seoul National University 1 Gwanak‐ro, Gwanak‐gu Seoul 08826 Korea; ^3^ Optical Nanoprocessing Lab Department of Mechanical Engineering Hanyang University 55 Hanyangdaehak‐ro, Sangnok‐gu Ansan 15588 Korea; ^4^ Intelligent Manufacturing System R&D Department Korea Institute of Industrial Technology 89 Yangdaegiro‐gil, Ipjang‐myeon, Seobuk‐gu Cheonan Chungcheongnam‐do 31056 Korea; ^5^ Institute of Advanced Machines and Design/Institute of Engineering Research Seoul National University Seoul 08826 Korea

**Keywords:** local rigidity modulation, mechanical dual mode, spatial micro‐water manipulation

## Abstract

Evolution has decided to gift an articular structure to vertebrates, but not to invertebrates, owing to their distinct survival strategies. An articular structure permits kinematic motion in creatures. However, it is inappropriate for creatures whose survival strategy depends on the high deformability of their body. Accordingly, a material in which the presence of the articular structure can be altered, allowing the use of two contradictory strategies, will be advantageous in diverse dynamic applications. Herein, spatial micro‐water molecule manipulation, termed engineering on variable occupation of water (EVO), that is used to realize a material with dual mechanical modes that exhibit extreme differences in stiffness is introduced. A transparent and homogeneous soft material (110 kPa) reversibly converts to an opaque material embodying a mechanical gradient (ranging from 1 GPa to 1 MPa) by on‐demand switching. Intensive theoretical analysis of EVO yields the design of spatial transformation scheme. The EVO gel accomplishes kinematic motion planning and shows great promise for multimodal kinematics. This approach paves the way for the development and application of smart functional materials.

## Graphical abstract

In this paper, a strategy that manipulates micro‐water of hydrogel in reversible, selective, and on‐demand manner is reported. It realizes instant switching between a transparent and homogeneously soft gel and an opaque and heterogeneously rigid solid with a monolithic mechanical gradient. These two mechanical dual modes mimicking the characteristic of invertebrate and vertebrate demonstrate multi‐modal kinematics.

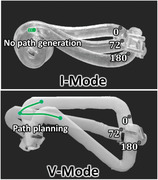

## 1 Introduction

Nature has driven creatures to adapt to their inherent mechanical environment and develop an optimum structure. Consequently, this has driven researchers to pursue nature‐inspired materials and applications. The optimum structures in nature are categorized into three classes based on their rigid characteristics. The first class is a homogeneously soft structure. Using the high shape adaptability of invertebrates, soft robot^[^ ^1^, ^2^, ^3^ ^]^ and gripper^[^ ^4^, ^5^, ^6^ ^]^ have been designed. The second class is a homogeneously rigid structure. For example, the side panel of Taipei 101^[^ ^7^ ^]^ was constructed by mimicking the structure of bamboo to enhance the lateral rigidity. The final group is a heterogeneously rigid structure. Linkage–joint system resembles an articular structure of vertebrate, which is specialized to kinematic motion. However, such artifacts function exclusively under specific circumstances because the bodies of different creatures have evolved from their distinctive environment. For instance, an octopus can easily hide in a rock crack, and bamboo can easily endure a static force, but not vice versa. There have been several attempts to use these two classes together by the advent of the homogeneous rigidity transformation.^[^ ^8^, ^9^, ^10^, ^11^, ^12^ ^]^ However, structures with heterogeneous rigidity could not be a member of the transformation group, despite its exclusive status in the mechanical functional structure classes.

The articular structure of vertebrates is composed of rigid bones linked to soft tissues. The skeletal muscle induces motion of the structure under controlled deformation, meaning that decisive strain only occurs at a relatively low rigidity region. The articular structure allows the structure to conduct kinematic motion by providing a finite degree of freedom (DOF) to the system. Moreover, bone and soft tissue are monolithically connected with a mechanical gradient to relax the stress concentration at the interface during dynamic operation.^[^ ^13^, ^14^ ^]^ Nature has successfully designed the mechanical gradient in the articular structure by introducing a gradient of water content.^[^ ^13^, ^15^ ^]^ Meanwhile, invertebrates have been designed to possess an evenly hydrated body with abundant water, giving them an entirely soft character.^[^ ^16^ ^]^ Therefore, a novel scheme that can be used to spatially manipulate micro‐water molecules would complete the rigidity transformation group.

Here, we demonstrate a spatial micro‐water molecule manipulation strategy for instant switching between a homogeneously soft state (invertebrate mode) and a heterogeneously rigid state with a monolithic mechanical gradient (vertebrate mode). The alternation of two mechanical modes is realized by a phase transition of supersaturated sodium acetate trihydrate (SAT) manipulating micro‐water molecules in poly(acrylamide/2‐acrylamido‐2‐methylpropane sulfonic acid) (P(AAm/AMPS)), which is a copolymer gel. Upon phase transition, the spatial distribution of micro‐water molecules within the copolymer evolves in a heterogeneous manner owing to the programmed molecular field distribution. Therefore, this transformation can be expressed as the engineering on variable occupation (EVO) of water molecules in a hydrogel. With the copolymerization ratio and volume fraction of the polymer as control parameters, the homogeneous rigidity of the invertebrate mode and the spatial rigidity changing ratio can be addressed. In particular, the ratio of change in water content, which plays a significant role in EVO is rigorously studied using a thermodynamic molecular field model. The four‐bar linkage model of the vertebrate mode demonstrates its appropriateness for kinetic and kinematic applications. Moreover, transformation within the heterogeneous rigidity group is realized by assigning kinematic multimodality to the vertebrate mode. Reprogramming the geometric parameters allows a single structure to realize multimodal motion‐planning. Using the unique phenomenon during EVO, we reliably connected a segment whose DOF is 1 to obtain a multiple‐DOF structure.

## 2 Switching between Vertebrate and Invertebrate

**Figure 1:**
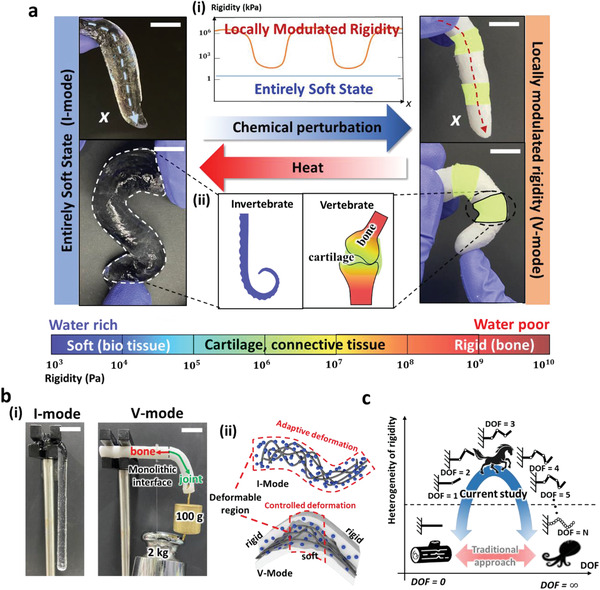
Reversible switching between soft structure and kinematic chain. a) Reversible switching between the models of tentacle and finger. (i) Spatial rigidity for each model measured along length direction (*x*‐axis). (ii) Equivalent structures in nature to EVO gel. Local rigidity is expressed according to the color bar below the schematics. b) (i) Statics of I‐mode and V‐mode. (ii) Microscopic distribution of water manipulator in each mode and relation between deformable region and distribution of water manipulator. c) The relation between heterogeneity of rigidity and kinematic DOF, and difference between EVO and traditional approach. All scale bars represent 2 cm.

As a salt *n*‐hydrate generally exhibits supersaturation behavior owing to its unique thermodynamic potential,^[^ ^17^ ^]^ it undergoes a phase transition only when a chemical perturbation is introduced. While the phase transition occurs, *n*‐water molecules are trapped in the crystal; these water molecules are then released from the crystal during the melting process. (Detailed discussion is in Note S1, Supporting Information.) Therefore, a set of molecules (anion, cation, and *n*‐water) can be interpreted as a water manipulator during the phase transition, and as the perturbation can be easily controlled, one can induce the reaction in a temporal, on‐demand manner (additional benefits of EVO are discussed in Notes S10 and S11, Supporting Information). Using these advantages, we developed a material that can reversibly modulate its spatial water distribution by applying sodium acetate tri‐hydrate (SAT), a water manipulator, to hydrogels whose mechanical properties are significantly dependent on the hydration amount.^[^ ^18^ ^]^ **Figure** **1**a shows the reversible transition for a finger model of the EVO gel, which shows a unique mechanical duality of the material. The *x*‐axis represents the length coordinate, and the color of the schematic corresponds to rigidity. Before transition, the appearance is completely transparent and the spatial rigidity is constant at ≈110 kPa along the *x*‐axis. We can address such a soft state as an invertebrate mode (I‐mode). The I‐mode is protected from undesired phase transitions by virtue of the chemical stability of the water manipulator. Moreover, a high mechanical compliance comparable to bio‐tissue allows the I‐mode to be used under circumstances requiring high shape adaptability, as illustrated in the left picture of Figure 1a. A single touch of the seed crystal induces the transparent finger to transform into a white finger, which has a spatially modulated rigidity distribution. Along the *x*‐axis, the rigidity fluctuates up and down in a range between 4.2 MPa and 1.09 GPa, as depicted in Figure 1a(i). This state is addressed as the vertebrate mode (V‐mode). We programmed the joints of the finger to transform into connective tissues (4.2 MPa), while other regions were transformed into bone‐like hard material (1.09 GPa). The decisive deformation occurs only at the connective region, which is similar to the mechanical response of a real finger. V‐mode can reversely transform into I‐mode when the temperature increases to 60 °C.

Statics of the I‐mode and V‐mode are shown in Figure 1b(i) and Figure S5 (Supporting Information). When the material in the I‐mode state was fixed at one end and suspended, the material appeared limp, verifying its homogenously soft character. In contrast, in the V‐mode state, which consists of a monolithically connected bone and joint structure, each region exhibited a distinctive response on applying static force. The bone endured 2 kgf without deformation, while the joint bent ≈4 cm when 100 gf was applied. Figure 1b(ii) schematically illustrates the microscopic discrepancy of water manipulators (blue spheres) and polymer strands (gray lines) between the two modes. The appropriate distribution of the swollen water manipulators induces an adaptive deformation in the I‐mode, whereas a heterogeneous water distribution similar to the real articular structure in the V‐mode generates controlled deformation in the middle region.

Figure 1c depicts the functional structures in nature with respect to heterogeneity of rigidity and kinematic DOF. Heterogeneity of rigidity generates a controlled deformation assigning regular (finite) DOF to a structure on a par with a leg of a horse. However, the structure inherently has a singular DOF (0 or infinity) in the absence of heterogeneity. In cases where kinematic motion planning is not essential, the singular DOFs have certain advantages. A zero‐DOF structure tends to maintain the shape of the structure without deformation under a high mechanical load, and an infinite DOF structure adaptably deforms under arbitrary loading, indicating high adjustability. So far, researchers have used singular DOFs by homogeneous rigidity transformation but have not addressed the transformation from singular DOF to regular DOF.

## 3 Engineering Mechanical Properties of Selectively Programmed Rigidity State

**Figure 2:**
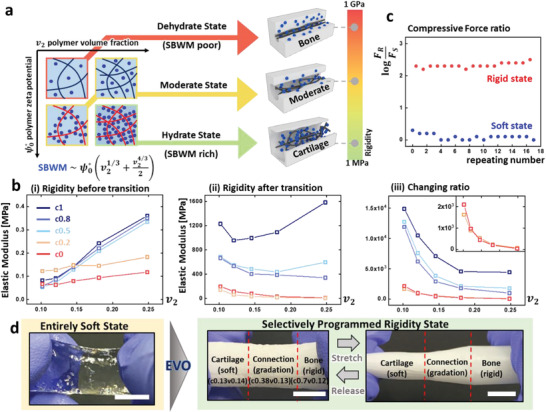
Engineering the spatial rigidity changing ratio. a) Illustration of strongly bounded water manipulator and its influence on the rigidity changing ratio. Blue spheres, strands, and white solid represent water manipulator, polymer chain, and crystal domain, respectively. b) Moduli of the component of EVO gel (i) before transition and (ii) after transition, and (iii) rigidity changing ratio. Inset is enlarged view for c0 and c0.2. All horizontal axes refer to polymer volume fraction, and each line corresponds to the copolymerization degree. c) Repeatability of EVO. d) Design of mechanical gradient kinematic pairs. All scale bars represent 1 cm.

We analyzed the strongly bound water manipulator (SBWM) to investigate factors that mainly affect EVO. SBWM is the initially swollen water manipulator in the region where the electrostatic field of the polymer strand effectively counteracts the entropic pressure of phase transition. **Figure** **2**a illustrates the influence of SBWM on rigidity after transition, and the reaction speed with respect to the amount of SBWM is shown in Figure S6 (Supporting Information). A system with a large amount of SBWM transits into a cartilage‐like state owing to the insufficient detachment of water manipulators near the polymer strands. However, with sufficient detachment, the gel with less SBWM transforms into a bone‐like state. We applied the Debye–Huckel model^[^ ^19^ ^]^ to a single cell of EVO gel to evaluate the electrochemical behavior of water manipulators near the strands. We scaled the dimensionless SBWM with polymer volume fraction *v*_2_, and the characteristic zeta potential *ψ* ^*^_0_ (A detailed derivation can be found in Note S4, Supporting Information)

(1) $$\mathrm{SBWM}=\frac{\bar{c}}{c_{0}}\frac{\bar{\xi }}{\xi }∼\psi_{0}^{*}\left(v_{2}^{1/3}+\frac{v_{2}^{4/3}}{2}\right)$$

where $\bar{c}\bar{\xi }$ is the strongly bounded water manipulator in a single cell, and *c*_0_ *ξ* is the total number of manipulators in the cell. Therefore, the degree of EVO, qualitatively related to SBWM, depends on two polymer variables, as described in Figure 2a. Engineering these two parameters simultaneously is essential because the rigidity before the phase transition and the changing ratio are monotonic functions of each dependent variable (Figure S8, Supporting Information). We modulated the polymer zeta potential by copolymerization of AAm and AMPS because AMPS has greater hydrophilicity than AAm due to the sulfonate group.^[^ ^20^, ^21^ ^]^ Moreover, the reactivity ratio of each monomer (*r*_AAm_ = 0.98, *r*_AMPS_ = 0.49)^[^ ^22^ ^]^ guarantees continuous manipulation of the zeta potential.

Figure 2b shows the elastic moduli before transition (i), after transition (ii), and the changing ratio between them (iii). Each curve represents the degree of copolymerization, and the horizontal axis represents the polymer volume fraction. We labeled the sample name cN1vN2, where N1 is a monomer fraction of AAm in a copolymer, and N2 is the polymer volume fraction. The sample name c0.5v0.25 represents equally copolymerized P(AAm‐AMPS) with a 0.25 polymer volume fraction. The moduli before transition monotonically increase with polymer volume fraction and the microstructure of the monomer affects the exponent of the curve.^[^ ^23^ ^]^ In contrast, the moduli after transition rely significantly on the interaction parameter. The moduli of the low interaction system (c1) increase non‐monotonically with volume fraction, while the moduli of the high interaction system (c0) behave in the opposite manner. Enlarging the interaction parameter causes the vertex of the U‐shape to move in the right direction. Furthermore, non‐monotonicity disappears under c0.5. The reason for such an unclear tendency is the distinctively different dependence between moduli before transition and SBWM. The modulus changing ratio is clearly arranged in a control plane, as predicted by SBWM, and it covers a wide range of changes. For instance, the modulus of c1v0.1 is increased 15 000‐fold after transition, while the modulus of c0v0.25 is increased 20‐fold. Armed with these values, we obtained a set of parameters that yields samples with rigidity of 0.15 MPa (c0.2v0.21, c0.5v0.15, c0.8v0.15) before transition and can instantly and reversibly transform to a material with rigidity of 45 MPa (cartilage), 350 MPa (connection), and 550 MPa (bone). The elastic property of the cartilage‐like state, which is appropriate for repeated deformation, is shown in Note S2 (Supporting Information). In addition, the reversibility of the EVO gel was characterized by measuring the force exerted when compressed to 1 mm. Moreover, the EVO gel maintained the mechanical properties of each mode during the 18 transition cycles, as shown in Figure 2c.

Figure 2d shows a rod‐shaped I‐mode that consists of three hidden components (c0.13v0.14 for cartilage, c0.38v0.13 for connection, and c0.7v0.12 for bone.). The completely transparent rod has homogeneous rigidity (0.15MPa), and heterogeneous rigidity emerges after transformation into V‐mode, as shown in the green box in Figure 2d. A mechanical gradient structure that is monolithically fabricated by diffusive polymerization (Note S6 and Figure S12, Supporting Information) appears when it is stretched. Heating the rod above 60 °C reversibly switches the EVO gel from V‐mode to I‐mode.

## 4 Micro‐Water Molecules Manipulation and Rigidity Changing Ratio

**Figure 3:**
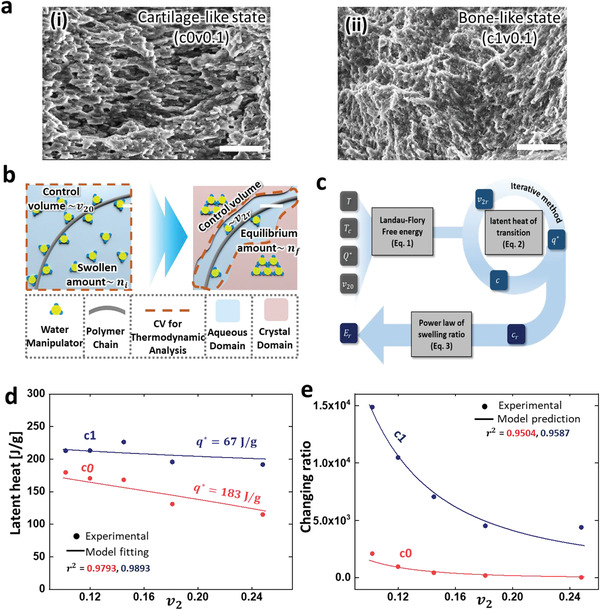
Thermodynamic analysis of EVO. a) ESEM image of crystal formation of (i) cartilage‐like state and (ii) bone‐like state. All scale bars represent 50 µm. b) Thermodynamic modeling describing electrochemical physics of unit cell in EVO gel. c) Schematic of the procedure to determine rigidity changing ratio. Gray color represents input parameters, dark blue color represents output parameter, and other colors represent intermediate variables. d) All horizontal axes refer to polymer volume fraction. Graph of latent heat of EVO gel to find the effective interaction energy. e) Results of model prediction of rigidity changing ratio.

**Figure** **3**a shows the environmental SEM (ESEM) images of c0v0.1 (i) and c1v0.1 (ii) after the transition. The crystal is uniformly formed near polymer strands in the small interaction system (ii), while the polymer in the huge interaction system (i) is surrounded by irregular and porous crystals. (Images for c1v0.25 and c0v0.25 are available in Figure S13, Supporting Information.) The proportion of SBWM was quantitatively evaluated as shown in the schematic model in Figure 3b. We considered only the transition from I‐mode to V‐mode because the thermodynamics of the transition is reversible. The gray strand depicts a single crosslinked unit, and a water manipulator is represented by tripods because it possesses the collective behavior of salt tri‐hydrates. Before the transition, water manipulators are freely floating in the liquid phase, and some interact with polymer strands, which is SBWM. We defined the macroscopically swollen amount of water manipulators as *n*_i_. During the transition, the entropic pressure is forced to solidify the manipulators, while the interaction energy q* prevents their transition to a solid. Consequently, water manipulators still remain in the liquid phase (*n*_f_) near the strand at the end of the reaction, as shown on the right side of Figure 3b.

We explored the change in Gibbs free energy with respect to the crystallization and solvent–gel interaction components. These factors counteract the free energy change because the hydration is favorable to the crystal and the polymer‐bound state, which implies that there is a stable point after transition. We constructed the proper free energy expressed by the two control parameters. (A detailed derivation can be found in Note S5, Supporting Information)

(2) $$\begin{array}{ccc} F-F_{0}\;\left(T,P\right) & = & n_{i}Q^{*}\left(1-\frac{T_{c}}{T}\right){\left(1-\frac{n}{n_{i}}\right)}^{2}\left[{\left(1-\frac{n}{n_{i}}\right)}^{2}-2\right]\\ & & +nkT\left[\log\left(1-v_{2r}\right)+\frac{q^{*}}{kT}v_{2r}\right] \end{array}$$

where *n* is the number of liquid manipulators, *v*_2_ *_r_* is the true volume fraction of the solvent varying with the liquid component *n*, q* is the effective interaction energy, and Q* and *T*_c_ are the latent heat and critical temperature of fusion of the sodium acetate trihydrate, respectively. The first term derived by the Landau scheme^[^ ^24^ ^]^ illustrates the characteristics of a pure manipulator system in which there are two extremums. It is necessary to consider mixing and interaction effects when introducing the polymer into the system. The second term, constructed using the Flory scheme,^[^ ^25^ ^]^ plays a field‐like role that breaks the symmetry of the initial pure manipulator system. As a result, it shifts the crystallization equilibrium point, initially given by *n*_i_, to *n*_f_ as shown in Figure S15b (Supporting Information). The high q* and large *v*_2_ induce a high intensity of the field, which leads to a greater shift of the equilibrium point. Thus, the field with a high intensity generates a cartilage‐like system because its proportion of SBWM largely deviates from that of the pure manipulator system (zero proportion of SBWM). The proportion of SBWM for a given field was evaluated by taking the derivative of the free energy with respect to *n*. To attain the only unknown parameter q*, we applied the fundamental thermodynamic relation of latent heat to Equation (2)

(3) $$L=T_{c}\Delta S=-q^{*}v_{2r}\left(1-c\right)-Q^{*}{\left(1-c\right)}^{2}\left[{\left(1-c\right)}^{2}-2\right]$$

Figure 3d shows the specific latent heat of c0 (red) and c1 (blue). As *v*_2_ increases, the latent heat of c0 gradually increases (35%), whereas the latent heat of c1 slightly decreases (10%). The thermodynamic effect of residual SBWM causes a lack of latent heat. We found the effective interaction energy using the regression (Note S3, Supporting Information) of Equation (3) to the latent heat: 67 and 183 J/g for c1 and c0, respectively (*r* ^2^ = 0.9893 for c0, 0.9793 for c1), which verifies that AMPS possesses threefold larger interaction energy than AAm due to the sulfonate group. As a result, c0 and c1 contain SBWM, water content changing ratio after transition, in the range of 15.5–31.4% and 8.0–12.4% in the prepared *v*_2_, respectively (Figure S15a, Supporting Information).

Because the mechanical modulus of the gel follows a scaling law with respect to the swelling ratio,^[^ ^18^ ^]^ the rigidity changing ratio should be a power function of the proportion of SBWM

(4) $$E_{r}/E_{s}={\left(\frac{n_{f}}{n_{i}}\right)}^{-\gamma }$$

where *E* is the modulus of each state. The subscripts r and s represent the rigid and soft states, respectively, and gamma is an exponent over 2.3 for the deswollen process.^[^ ^18^ ^]^ Figure 3e shows the modulus changes of each state with model prediction, assuming the gamma value as 3.81. We believe that the suitable gamma value appears 1.66‐fold larger than that of a pure gel because crystals are developed outside of the coagulated polymer strands. This scheme, based on micro‐water molecule manipulation, describes the rigidity changing ratio (*r* ^2^ = 0.9587 for c0, 0.9504 for c1).

## 5 Kinematic Motion Planning of EVO Gel and its Expandability

**Figure 4:**
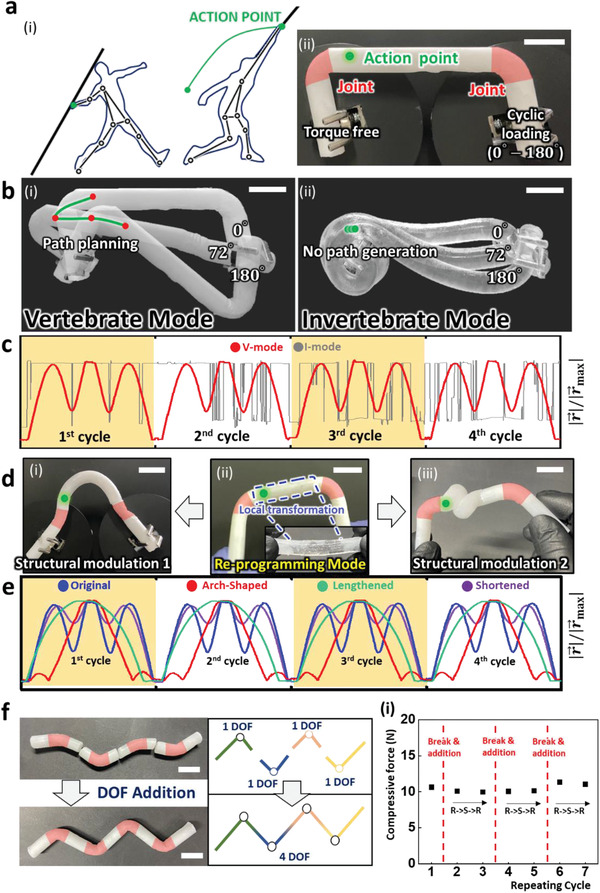
Kinematics and expandability of EVO gel. a) Illustration of trajectory of action point in kinematic chains and model kinematic chain of V‐mode. The green dot represents the action point, and repeated torque is applied to the right pivot. b) Influence of the articular structure on kinematic motion planning of action point. c) Normalized displacement of action point inferring controllability and repeatability of V‐mode compared to I‐mode. d) Re‐programming of geometric parameters for generating multi‐modal trajectory. The original kinematic chain (ii) is reprogrammed to arch (i) and twisted (iii). e) Normalized displacement of action point with original, arch, lengthened, and shortened shape. f) Schematic of DOF addition and (i) its reliability graph. The red dotted line corresponds to the break and addition process. Between additions, transformation of R‐S‐R was applied. All scale bars are 2 cm.

**Figure** **4**a(i) illustrates an athlete generating a precise, efficient, and repetitive trajectory of force while throwing the javelin to a great distance. A kinematic chain embodied in an athlete is capable of generating an efficient trajectory.^[^ ^26^, ^27^ ^]^ Because the hand transmitting force to the javelin works as an action point, the trajectory made by the action point becomes the key parameter in javelin throwing. Therefore, to verify the applicability of V‐mode as a kinematic chain, we considered a four‐bar linkage model of the V‐mode, as shown in Figure 4a(ii). The red parts are kinematic pairs connecting solid bodies monolithically in a mechanical gradient manner. The motor generates a cyclic rotation of the right pivot in the range between a 0° and 180° angle, while the left pivot is torque‐free.

Figure 4b shows the kinematic operation in V‐mode (i) and I‐mode (ii) (the real‐time operation can be found in Video S1, Supporting Information). The kinematic pairs of the V‐mode efficiently carried out the structural deformation, which is their function in a dynamic operation. Such an appropriate controlled deformation drives the V‐mode to produce a clear and distinct trajectory of the action point, as shown by the green line in Figure 4b(i). In contrast, the same mechanical input could not generate any path of the action point in I‐mode, where the entire deformation only occurred near the input pivot, as shown in Figure 4b(ii). The trajectory of the action point was further investigated by analyzing the normalized displacement of the action point, $|\vec{r}\left|/\right|{\vec{r}}_{\max}|$, which represents the distance from the starting point to the action point. The normalized displacements of the V‐mode and I‐mode are shown in Figure 4c. In the case of I‐mode, irregular and unpredictable displacements were obtained, showing no controllability and repeatability. In contrast, the V‐mode demonstrated three distinct humps on a single operation, and perfect repetition was achieved through four cycles. (Note that the extremums in the normalized displacement of the V‐mode are indicated by red dots in Figure 4b(i)) It implies that the V‐mode possesses the exceptional features of an articular structure, whereas the I‐mode can adaptably wrap around the left pivot.

Furthermore, structural modulation and DOF addition allowed the EVO gel in V‐mode to have kinematic multimodality. We applied EVO to link 2 in Figure 4d(ii) to perform structural modulation (detailed procedure in Note S8, Supporting Information). The EVO gel was modulated into arbitrary shapes, including arch‐shaped (Figure 4d(i)), lengthened (Figure S17, Supporting Information) and shortened shapes (Figure 4d(iii)). Real‐time operation of amended states is shown in Video S2 (Supporting Information). Figure 4e exhibits the normalized displacement of the action point for each modulated one. Compared to the one in Figure 4a(ii), the arch‐shaped and the lengthened ones generated a monotonically increasing displacement in the first half cycle. The shortened one generated three smaller humps than the original one, meaning that it could be utilized for modulating force exertion, as shown in Figure S17 (Supporting Information). However, despite the variable‐manipulated paths, the repeatability was safely guaranteed, as shown in Figure 4e. DOF addition can be achieved by the micro‐deswollen effect, which expands the mechanical functionality of EVO (further explanation for the mechanism is discussed in Note S9, Supporting Information). We successfully attached four different single DOFs to obtain a 4‐DOF kinematic chain, as shown in Figure 4f. Moreover, Figure 4f(i) shows the force needed for 1 mm compression during seven repetitive addition cycles, where the red line indicates the breakage‐and‐addition process. The additive part exhibited similar mechanical property as that of the original unbroken one, indicating a reliable connection of DOF addition.

## 6 Conclusions

In summary, we presented a spatial micro‐water molecule manipulation method for switching between vertebrate (V‐mode) and invertebrate (I‐mode) states. The transformation between the V‐mode and I‐mode is realized by manipulating the SBWM, which directly depends on the degree of the interaction energy between the polymer and water manipulator. The operating physics was rigorously investigated by considering the combined Landau‐Flory free energy, and a correlation between the dominant parameters and the rigidity changing ratio was found. We demonstrated that the V‐mode carries great controllability and repeatability by inspecting the normalized displacement of the action point. Furthermore, kinematic multi‐modality was assigned to the V‐mode by structural modulation and DOF addition. It is also expected that attaching actuators to each kinematic pair of structurally remarkable EVO gels will contribute to future generation robots that operate under various mechanical environments.

## 7 Experimental Section

### Materials

Acrylamide (suitable for electrophoresis, ≥99%), 2‐acrylamido‐2‐methyl‐1‐propanesulfonic acid (99%), N,Nʹ‐methylenebis(acrylamide) (99%), ammonium persulfate (ACS reagent, ≥98.0%), and sodium acetate (anhydrous, ReagentPlus, ≥99.0%) were purchased from Sigma Aldrich. All chemicals were used without further purification.

### Preparation of Precursor

Precursor of EVO gel was prepared by dissolving monomer (AAm and/or AMPS), cross‐linker (N,N′‐methylenebis(acrylamide)) in the supersaturated solution of SAT. The weight ratio between sodium acetate and water was 13:10 which was optimized for supersaturation behavior and 1:3 mol ratio between them for water manipulation. The weight ratio between monomer of AAm and AMPS was represented as a monomer fraction of AAm in a copolymer, *c*. The weight ratio between total monomer and the cross‐linker was 1:0.0007 for AAm and 1:0.0037 for AMPS. The weight ratio between total monomer and SAT was distinguished by the polymer concentration, *v*_2_. After the precursor solution became homogeneous, an initiator (ammonium persulfate) was added to the solution. The weight ratio of the monomer to the initiator was 1:0.0045.

### Fabrication Process for the Kinetic Bar and the Finger‐Shaped EVO Gel

Schematics of fabrication procedure of kinematic bar and finger model are in Figures S3 and S4 (Supporting Information), respectively. The fabrication process may consist of several steps depending on the number of bone and cartilage structures. First, bone precursor (c1v0.11) was poured to the mold. Then, the halfway polymerizing step was conducted at 60 °C for 60 min considering duration time for complete gelation (90 min). After that, the diffusing step took place in room temperature for 30 min allowing the next cartilage precursor (c0v0.25) to diffuse into the halfway polymerized gel. These two steps were conducted until the final bone precursor (c1v0.11) is poured. Finally, the last step was conducted at 60 °C for 90 min for the complete polymerization.

### Measurement of Elastic Modulus and Latent Heat

The elastic modulus was measured using the micro‐indentation method with a universal testing machine (Instron 5948 Mircrotester). Gels with various control parameters were fabricated in a Petri dish. They were compressed by a universal testing machine with an indentation tip of radius of 5.25 mm. The experiment detail is presented in Note S7 (Supporting Information). The latent heat of the EVO gel was measured using a differential scanning calorimeter (DSC, Discovery DSC, TA Instruments). The temperature was increased from 20 to 90 °C with a 5 °C min^−1^ ramp. Latent heat was evaluated by integrating the heat flow in the above temperature range. The measurement results for the overall control parameters are shown in Figure S14 (Supporting Information).

### Cyclic Compressing Force Test of EVO Gel

The EVO gel was made in a customized mold (Figure S11, Supporting Information). The size of the gel was 40 mm × 40 mm and encapsulated by Ecoflex to prevent the gel from drying. The experimental setup is shown in Figure S11 (Supporting Information) and the formula of the precursor is c1v0.11. It was compressed for 3 mm with custom‐made force measurements (Load Cell, Futek), and the force was measured 60 s after the compression. Compressive force of the initially prepared I‐mode gel was tested and transformed into V‐mode by a touch of seed. Then compressive force of the V‐mode gel was tested and transformed into I‐mode at 60 °C for 90 min. This measurement was repeated ten times.

### Cyclic Compressing Force Test of EVO Gel for Reliability of DOF Addition

The same mold for cyclic compressing force test was used. The gel in V‐mode was compressed for 1 mm with custom‐made force measurements and the force was measured 60 s after the compression. Before heating the sample at 60 °C for 90 min, it was broken into two pieces right in the middle. Then a chemical perturbation was given after the gel became the I‐mode to transform it into the V‐mode. Then, the V‐mode gel was measured again. After measurement, the sample transformed to the I‐mode at 60 °C for 90 min. The chemical perturbation was given again after the sample became the I‐mode. Then again, the sample was measured and broken. This was repeated three times. The formula of the precursor is c1v0.11.

## Conflict of Interest

The authors declare no conflict of interest.

## Author Contributions

I.H. and M.K. contributed equally to this work. I.H., M.K., and S.H.K. conceived and initiated the study. I.H. and M.K. designed and performed the experiments. I.H. and M.K. constructed the theory and analyzed the data. S.H.K. supervised the research program. K.K.K. and S.H. advised on the development of the idea. All authors discussed the results and wrote the manuscript.

## Supporting information

Supporting Information

Supplemental Video 1

Supplemental Video 2

## Data Availability

The data that support the findings of this study are available from the corresponding author upon request.
