# Supplementary material for: Reversible, Selective, Ultrawide‐Range Variable Stiffness Control by Spatial Micro‐Water Molecule Manipulation
Source: Adv Sci (Weinh). 2021 Aug 27;8(20):2102536. doi: 10.1002/advs.202102536 (PMC8529442; doi:10.1002/advs.202102536)
Supplement: Supplementary file 1 — Supporting Information [file ADVS-8-2102536-s001.pdf]

© 2021 Wiley-VCH GmbH

ADVANCED SCIENCE
Open Access

Supporting Information

for *Adv. Sci.,* DOI: 10.1002/advs.202102536

Reversible, Selective, Ultrawide-range Variable Stiffness Control by Spatial Micro Water Molecule Manipulation

*Inho Ha, Minwoo Kim, KyunKyu Kim, Sukjoon Hong, Hyunmin Cho, Jinhyeong Kwon, Seonggeun Han, Yeosang Yoon, Phillip Won, and Seung Hwan Ko**

Supplementary Information

Reversible, Selective, Ultrawide-range Variable Stiffness Control by Spatial Micro Water Molecule Manipulation

Inho Ha[1,2]†, Minwoo Kim[1,2]†, KyunKyu Kim[1,2], Sukjoon Hong[3], Hyunmin Cho[2], Jinhyeong Kwon[4], Seonggeun Han[2], Yeosang Yoon[2], Phillip Won[2], and Seung Hwan Ko[1,2,5]*

[1] Soft Robotics Research Center, Seoul National University, 1 Gwanak-ro, Gwanak-gu, Seoul 08826, Korea
[2]Applied Nano and Thermal Science Lab, Department of Mechanical Engineering, Seoul National University, 1 Gwanak-ro, Gwanak-gu, Seoul 08826, Korea
[3]Optical Nanoprocessing Lab, Department of Mechanical Engineering, Hanyang University, 55 Hanyangdaehak-ro, Sangnok-gu, Ansan 15588, Korea
[4]Intelligent Manufacturing System R&D Department, Korea Institute of Industrial Technology, 89 Yangdaegiro-gil, Ipjang-myeon, Seobuk-gu, Cheonan, Chungcheongnam-do 31056, Korea
[5] Institute of Advanced Machines and Design/ Institute of Engineering Research, Seoul National University, Seoul 08826, Korea

[*] To whom correspondence should be addressed.
E-mail: maxko@snu.ac.kr

[†] Inho Ha and MinwooKim contributed equally to this work.

Supplementary Figures

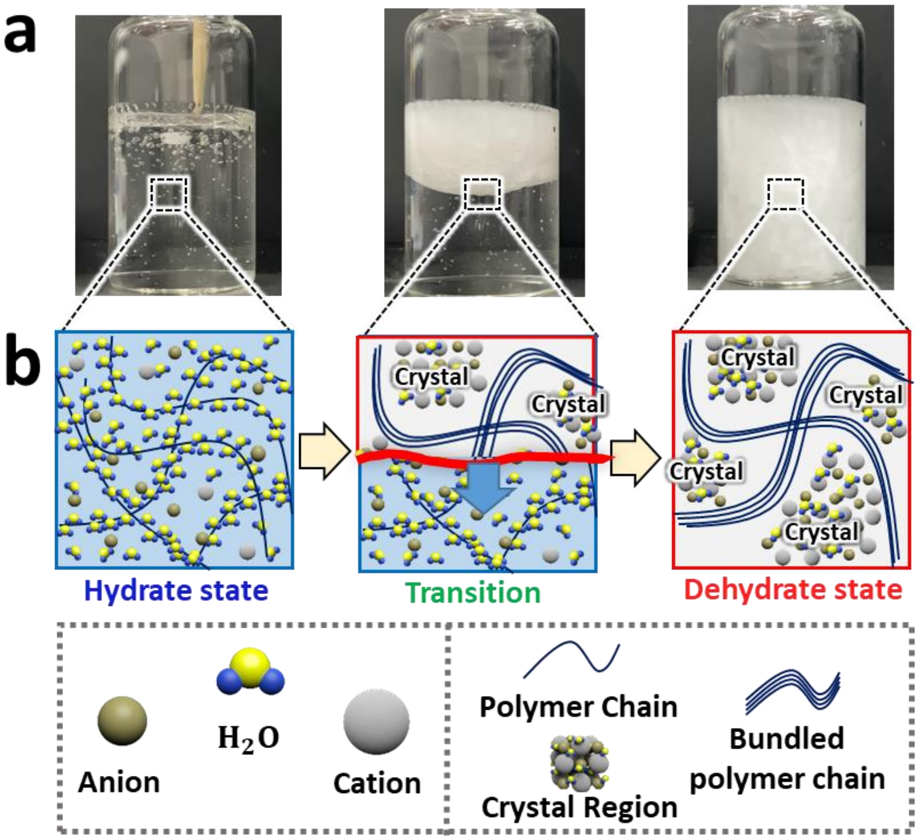

Supplementary Figure 1 | Micro structure of EVO gel during transition. a. optical image of cartilage-like gel during transition. Scale bars are 1cm. **b.** Corresponding micro structure considering water manipulators and polymer chains.

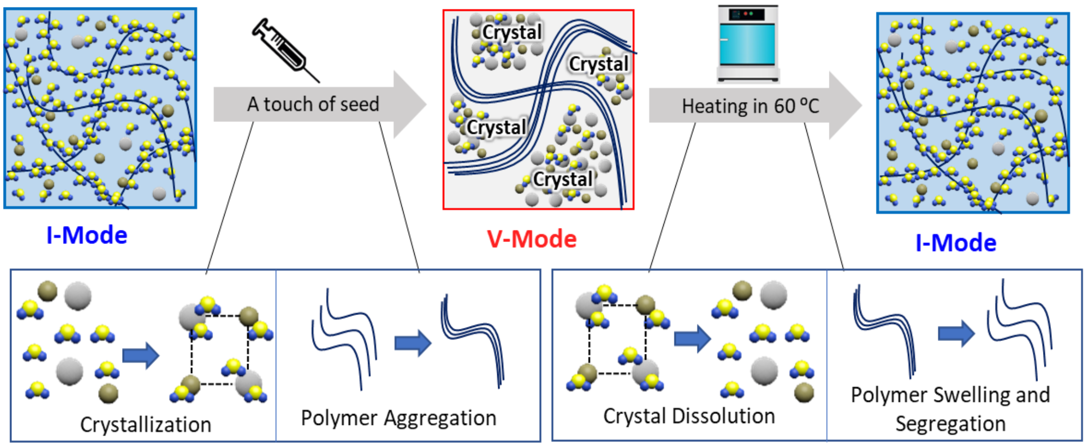

Supplementary Figure 2| Mechanistic reversibility of EVO. Crystallization induced polymer aggregation in transition from I-mode to V-mode and crystal dissolution induced polymer swelling and segregation in opposite transition

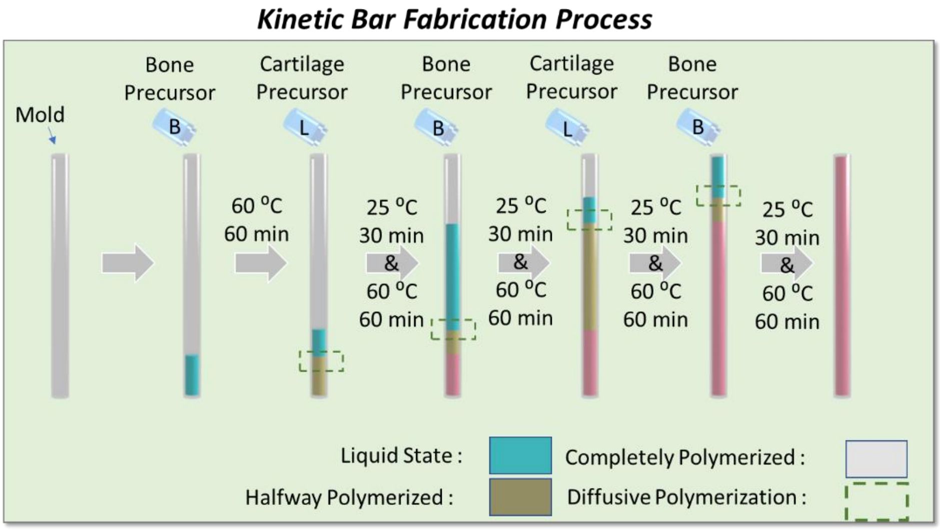

Supplementary Figure 3| Fabrication process of kinetic bar

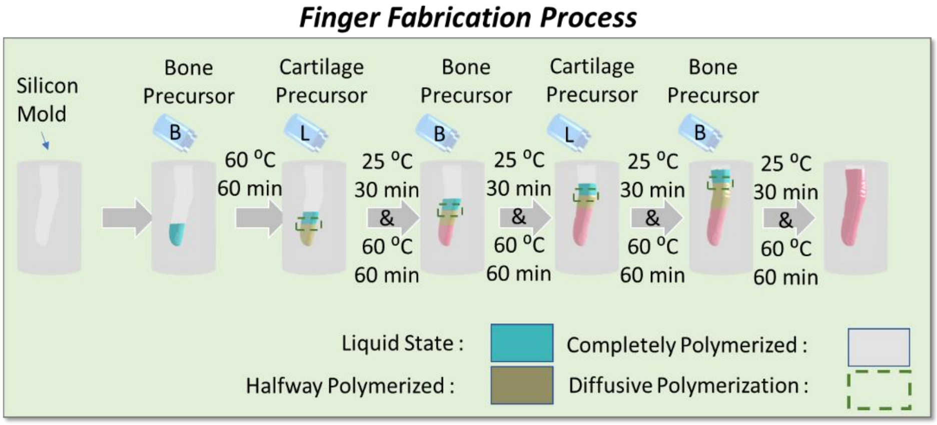

Supplementary Figure 4| Fabrication process of finger model

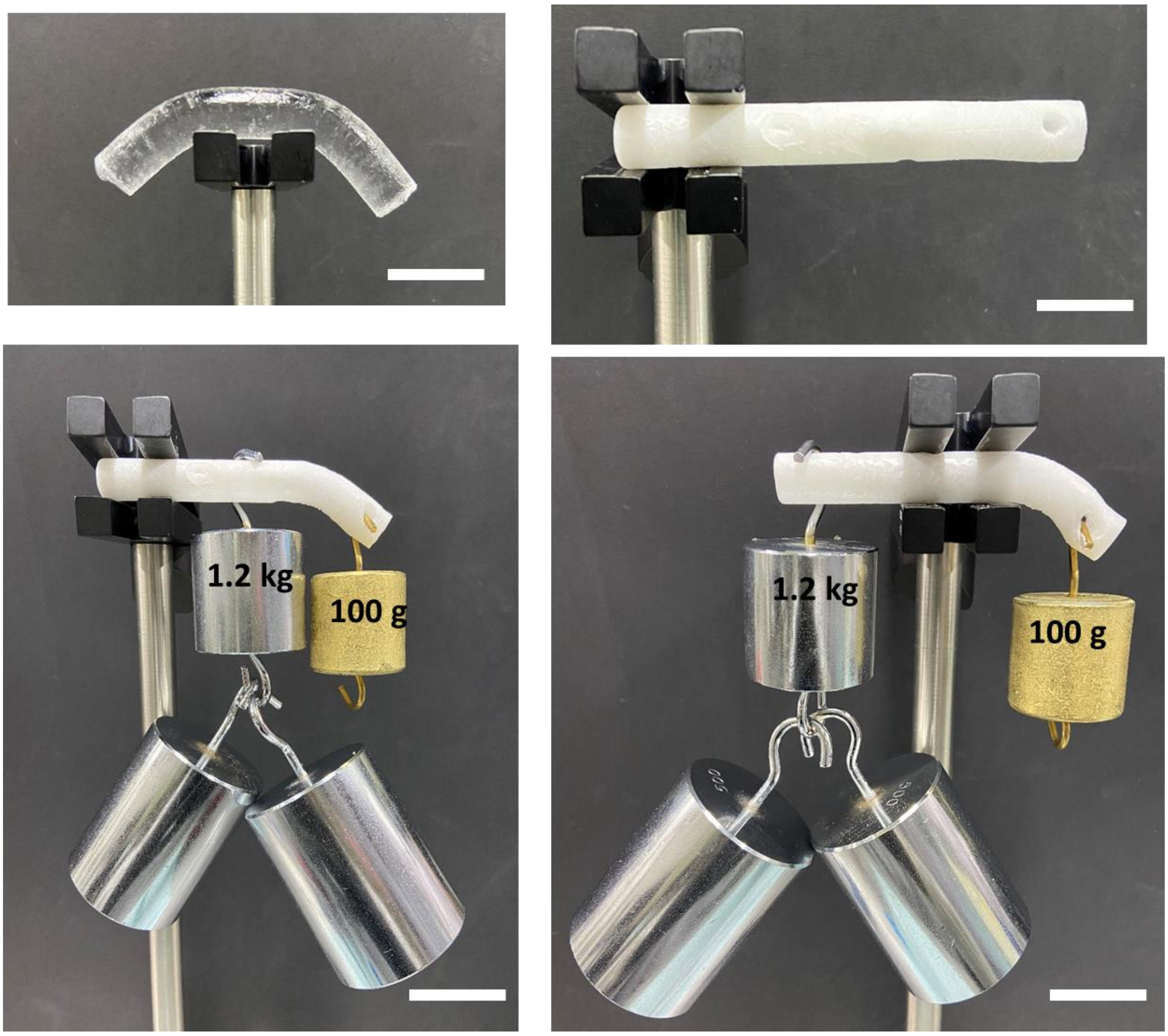

Supplementary Figure 5 | Statics of EVO gel for various configuration scale bars are 2 cm.

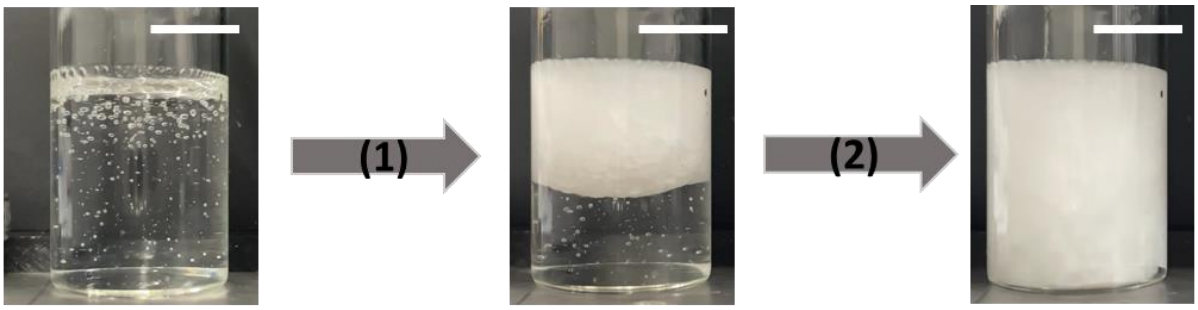

	Time for (1)	Time for (2)	Reaction speed
c0	57 sec	128 sec	0.20 mm/s
c1	7 sec	15 sec	1.73 mm/s

Supplementary Figure 6 | Reaction speed regarding polymer interaction. Scale bars are 1 cm.

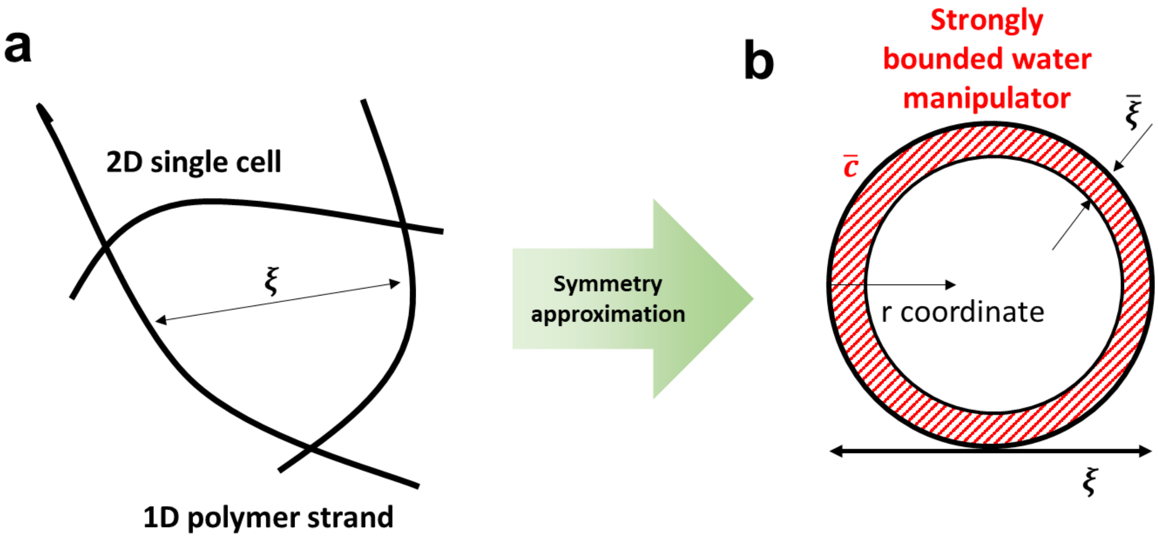

Supplementary Figure 7 | Symmetric approximation of single cell of EVO gel. a. 2D single cell surrounded by 1D polymer strand. ξ denotes the size of cell. **b.** Symmetric approximated cell. Bar variables infers the SBWM quantities.

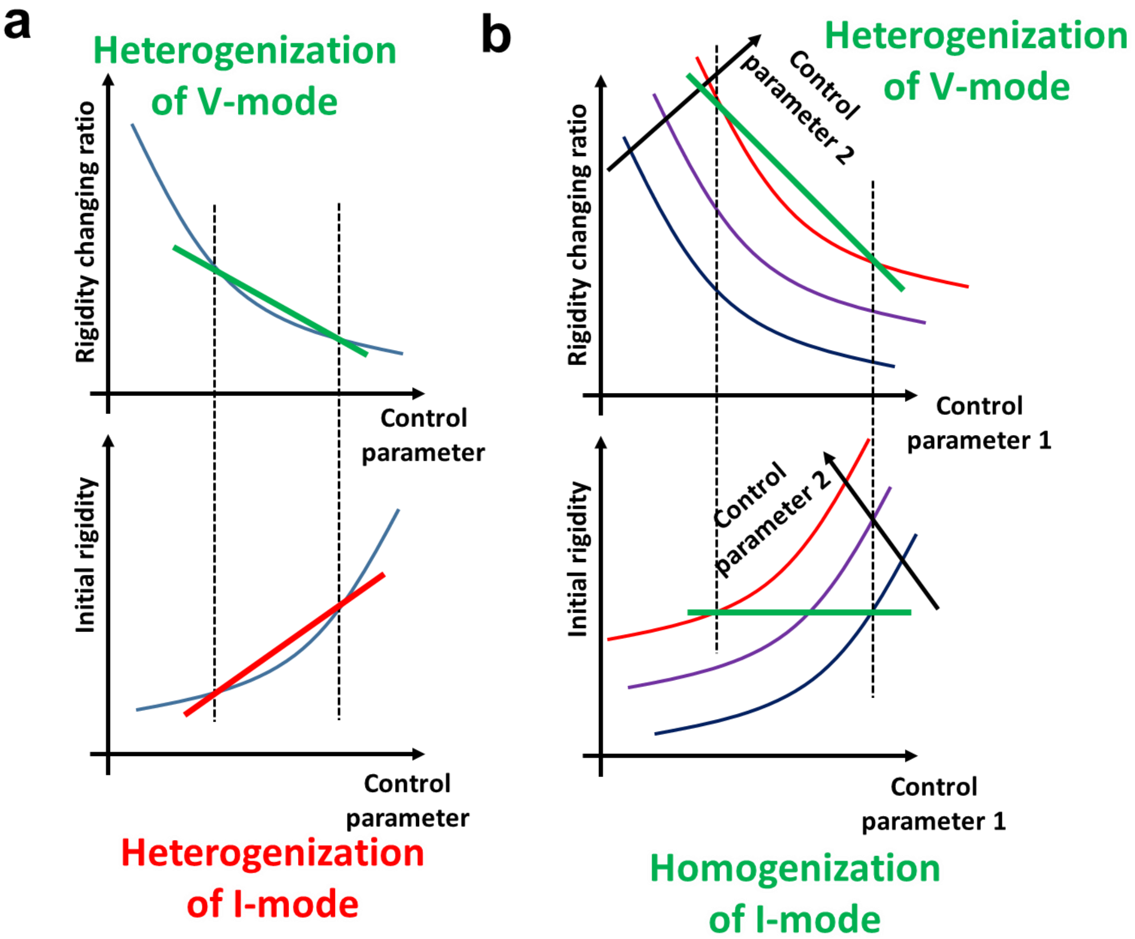

Supplementary Figure 8 | Necessity of multi control parameters for EVO. a. Rigidity transformation scheme with single control parameter **b.** Rigidity transformation scheme with two control parameters

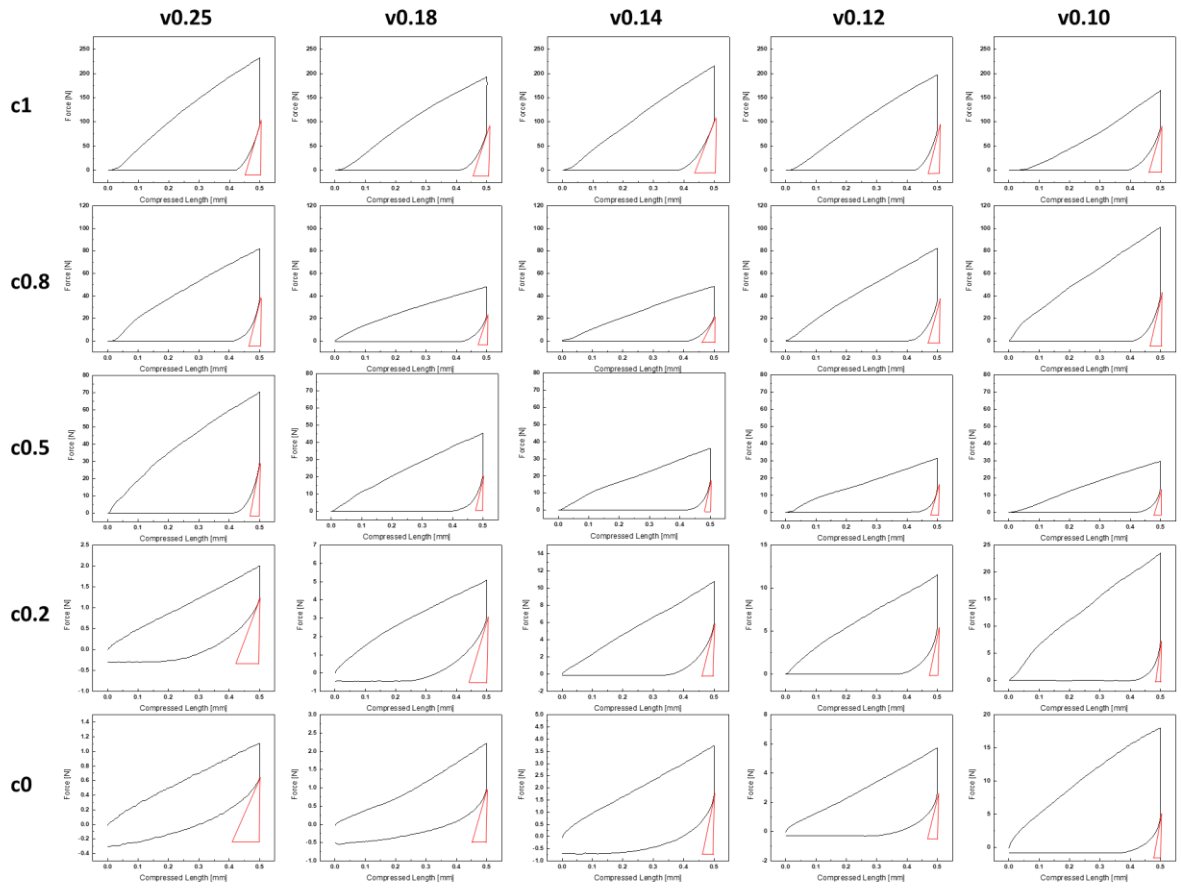

Supplementary Figure 9 | Micro-indentation results for various control parameters of EVO gel.

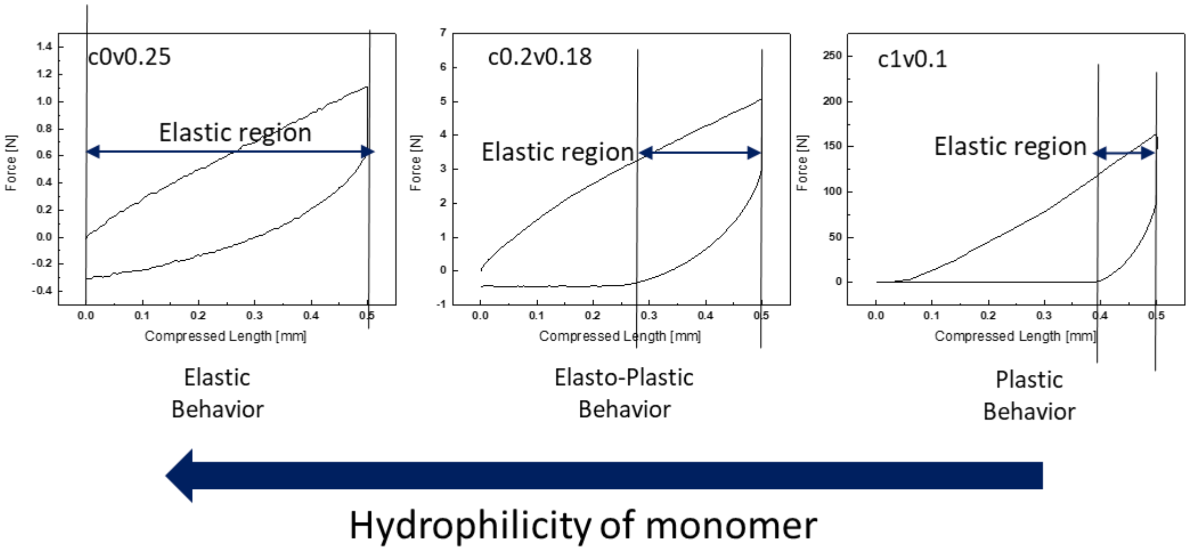

Supplementary Figure 10 | Tendency of the elastic region regarding the hydrophilicity of monomer.

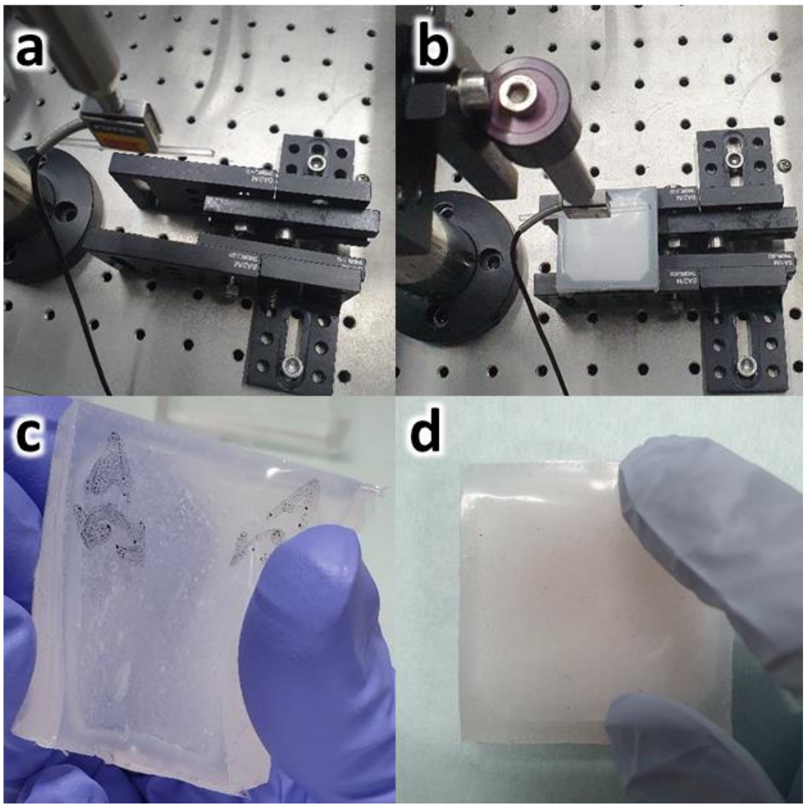

Supplementary Figure 11 | Experimental setup for repeated compression test. a. Before loading a sample. **b.** After loading a sample. **c.** A sample in hydrated mode. **d.** A sample in dehydrated mode

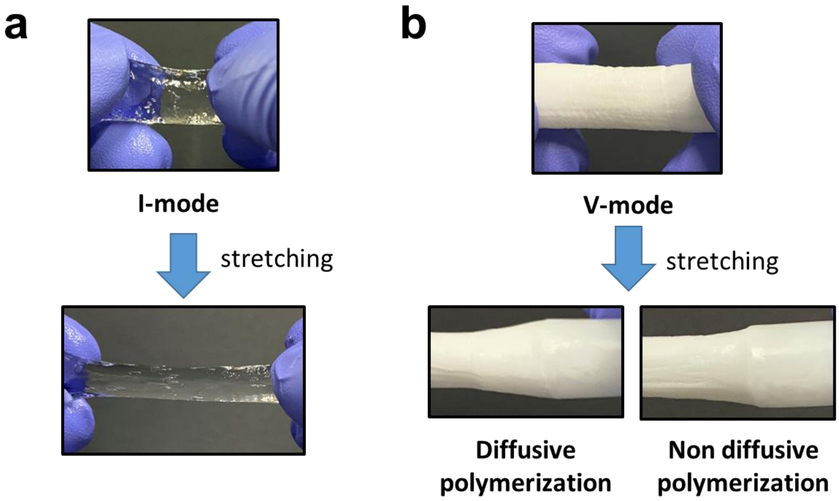

Supplementary Figure 12 | Diffusive polymerization for mechanical gradient in V-mode.
a. Stretching of I-mode. **b.** Stretching of V-modes with differently polymerized method.

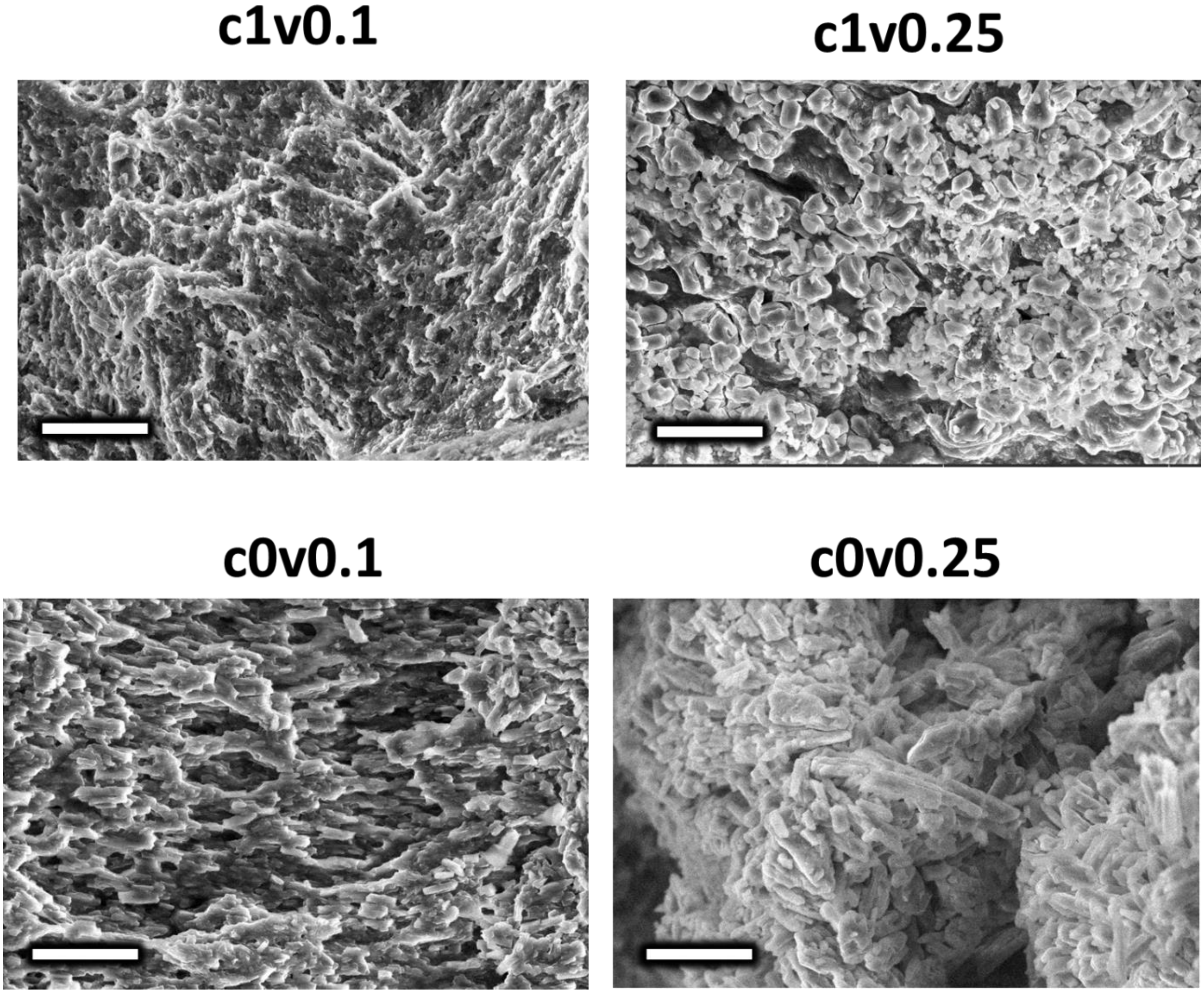

Supplementary Figure 13 | ESEM images of the EVO gel in various composition. (c1v0.1, c1v0.25, c0v0.1, c0v0.25) Scale bars are 50 μm.

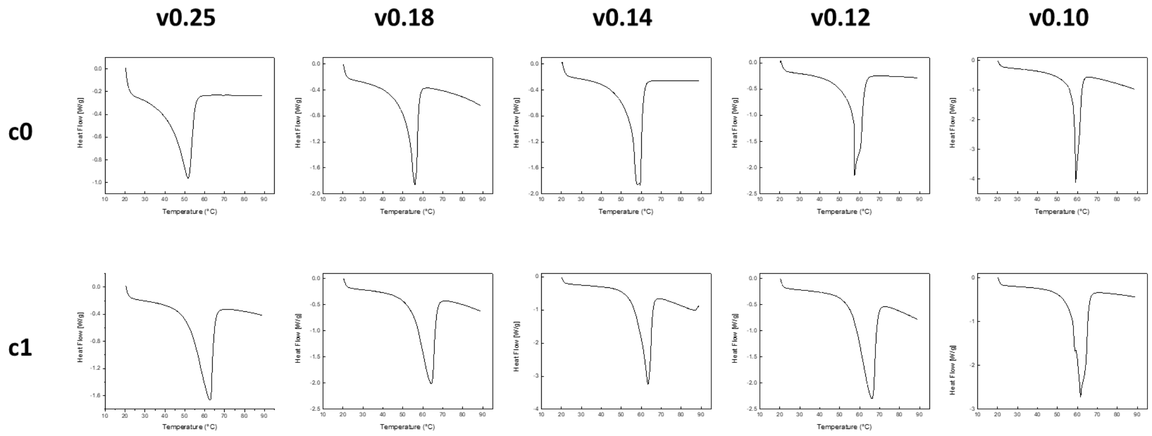

Supplementary Figure 14 | | Differential Scanning Calorimeter (DSC) measurement results for overall control parameters.

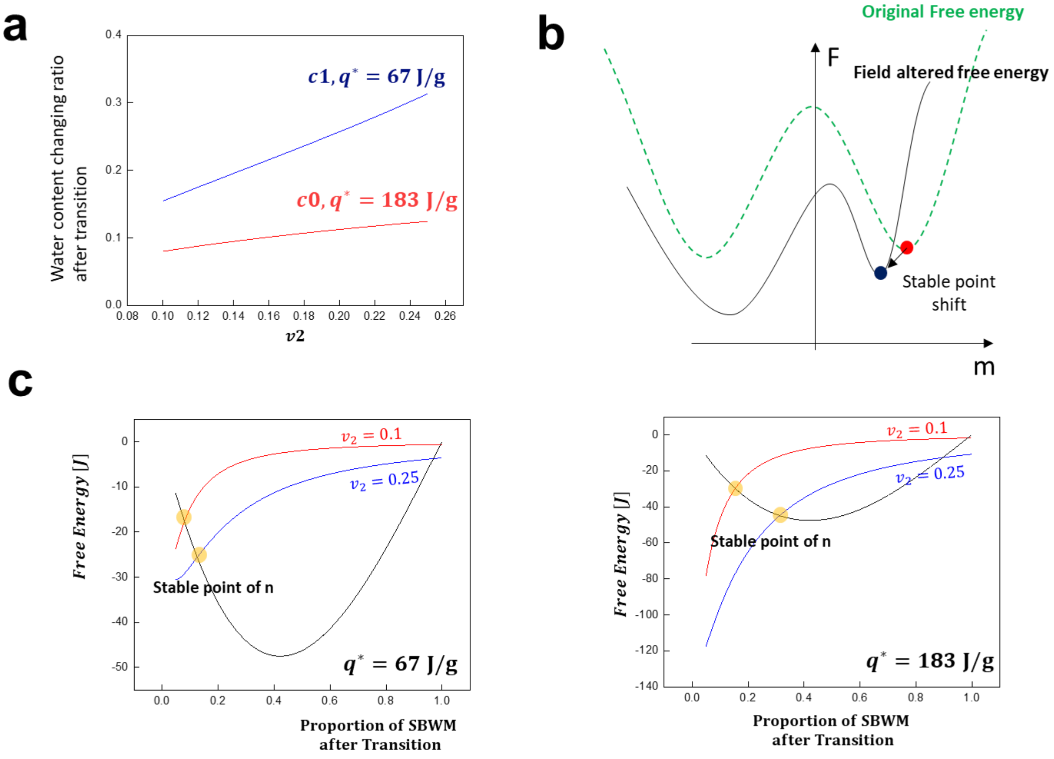

Supplementary Figure 15 | Thermodynamic field consideration of EVO. a. Watercontentchangingratioaftertransitionfor c0 and c1. **b.** Symmetry breaking on free energy by field. **c**. Equilibrium point of SBWM after transition for c1 and c0.Black lines are pure manipulator system and the colored curves are the molecular field components.

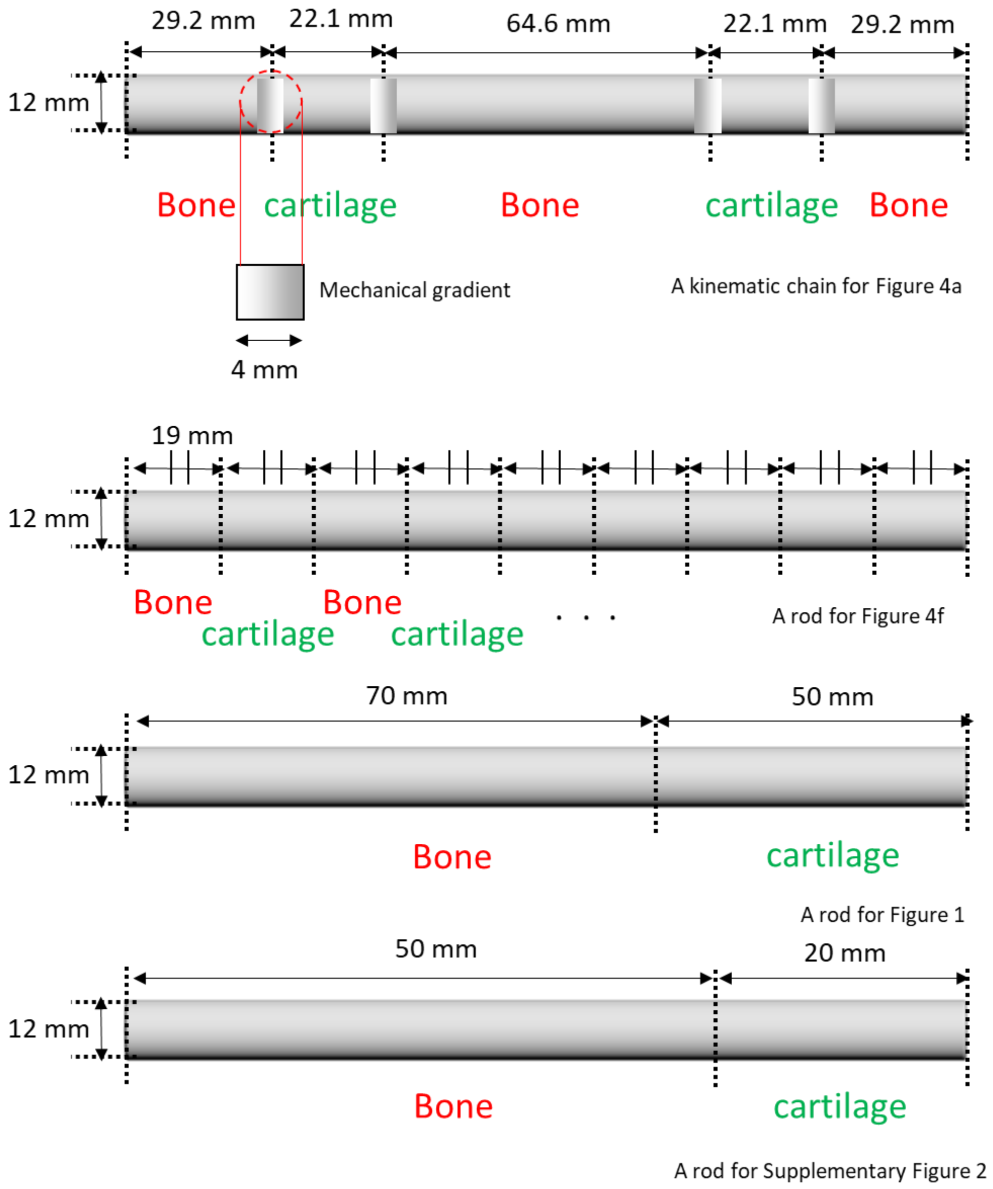

s

Supplementary Figure 16 | Geometric parameters of kinematic chain model and rods. a. A kinematic chain for Figure 4a. **b.** ArodforFigure4f.**c.**ArodforFigure1.**d.**ArodforSupplementaryFigure2.

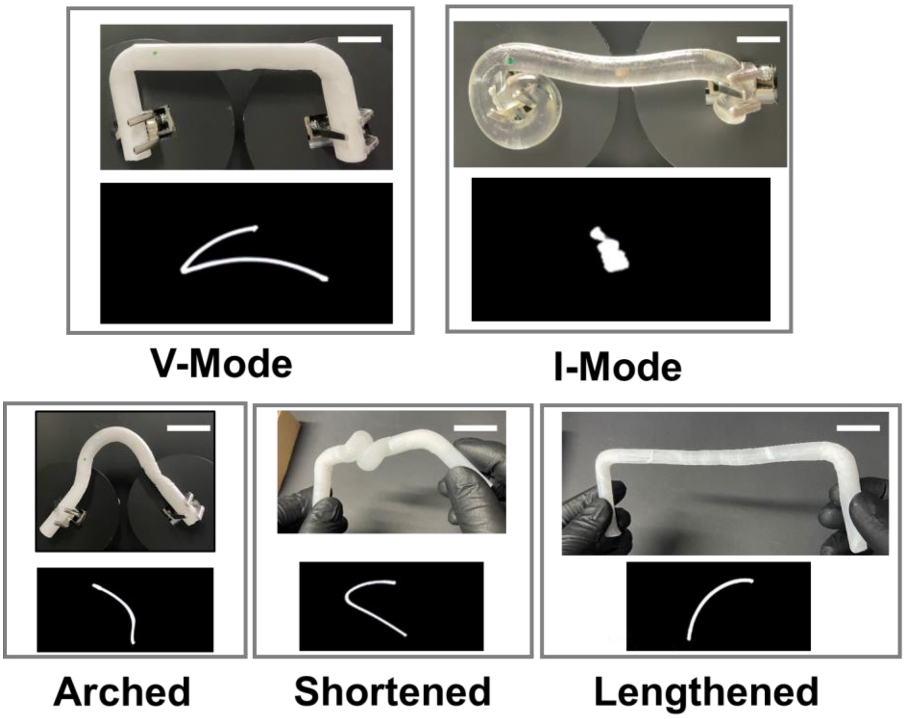

Supplementary Figure 17| Trajectory of action point of various kinematic chains and multimodality of EVO gel. Scale bars are 2cm.

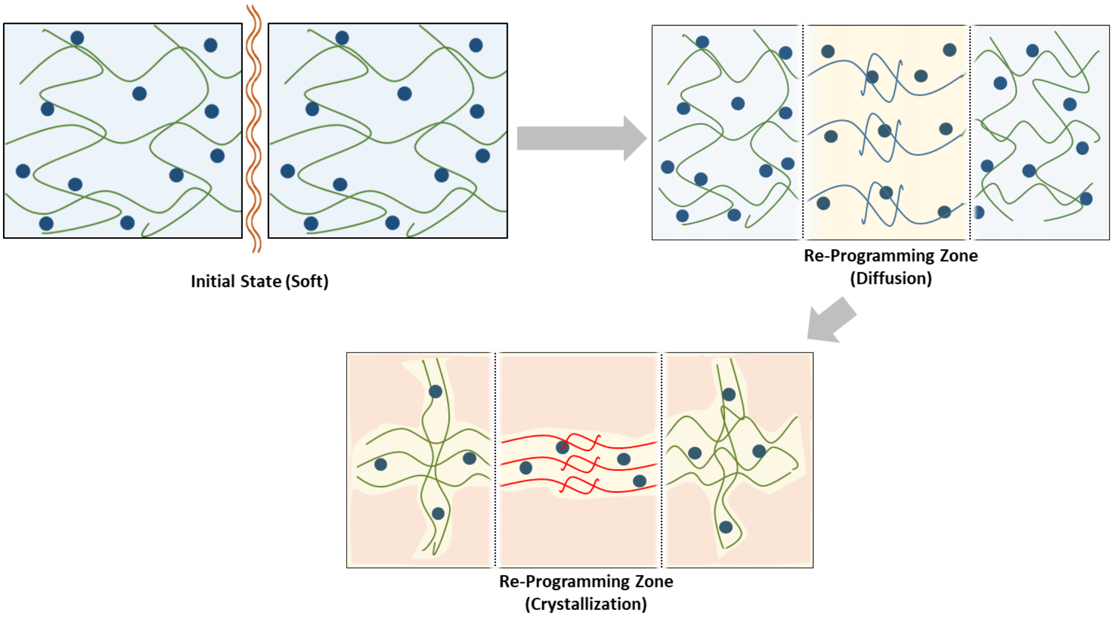

Supplementary Figure 18 | Mechanism of DOF addition process.

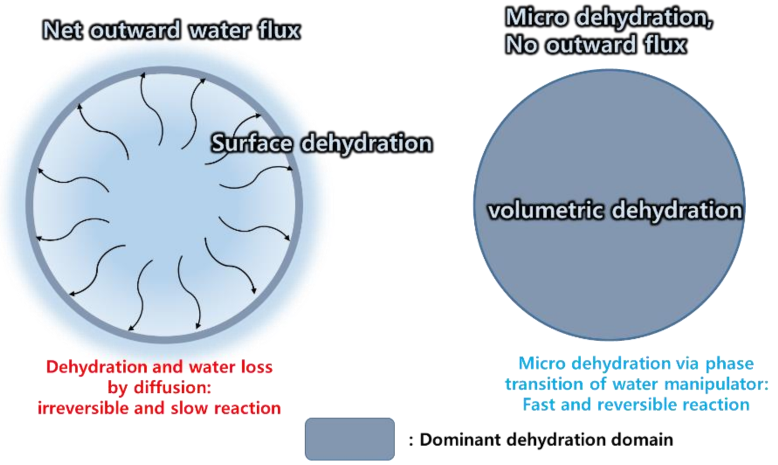

Supplementary Figure 19 | Comparison between EVO and evaporation. Characteristic of strengthening of hydrogel with respect to domain of dehydration

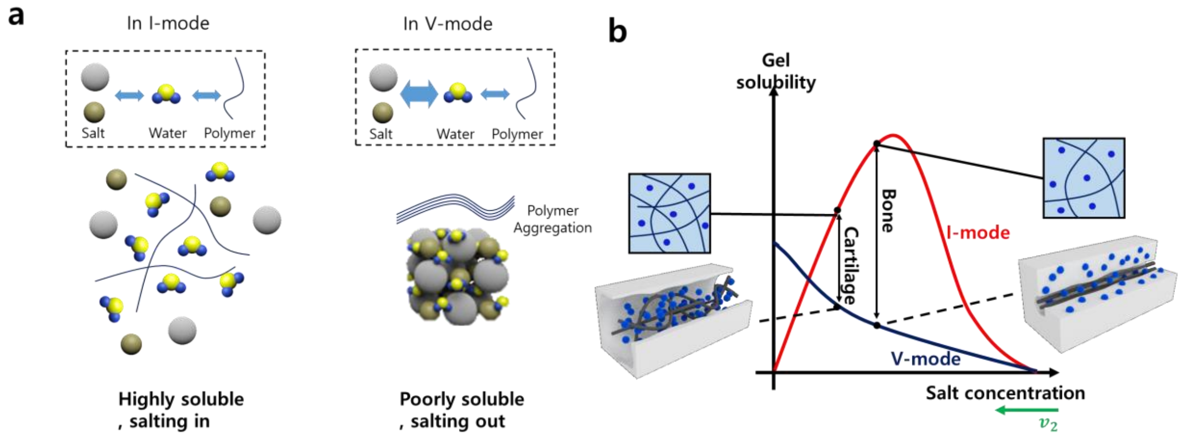

Supplementary Figure 20 | Mechanistic analogy between EVO and salting-out. a. The component that mainly interacts with water molecules depending on the phase of salts and its impact on polymer solubility.**b.** Mechanistic interpretation of EVO in terms of salting out.

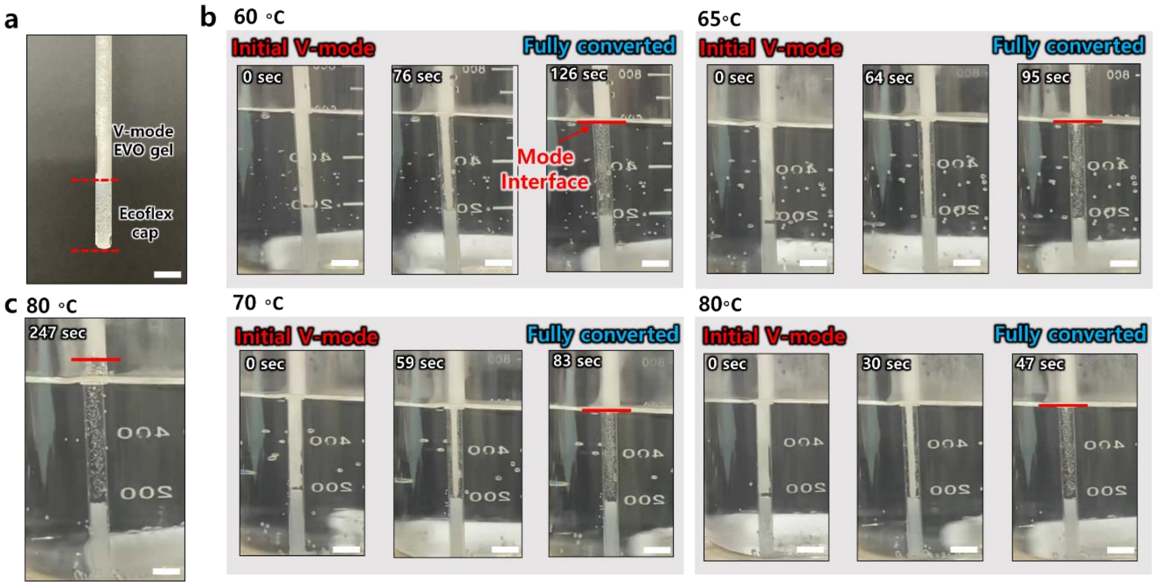

Supplementary Figure 21 | Controllability of local amending process. a. V-mode EVO gel covered by glass cylinder and sealed with Ecoflex to prevent invasion of water. **b.** Transient change of bone EVO gel when locally heated (60, 65, 70, 80 ˚C). **c.** Equilibrium state of mode interface in 80 ˚C when locally heated. All scale bars are 1cm.

Supplementary Tables

**Physical and geometric properties in Equation S29, S30**		**Values**	**References**
Thermal conductivity of V-mode	Thermal conductivity of SAT	0.7 W/m·K	Dannemand*et. al.* Solar Energy Materials and Solar Cells, 2016.
	Thermal conductivity of PAAm	0.56 W/m·K	Davidson *el. al.* International Journal of Hyperthermia, 2003
Conduction length, *L*		1 cm	experimental
Conduction area, *A*		1 cm^2	experimental
Temperature difference, ΔT		40 K	experimental
Conduction time, Δt		100 sec	experimental
Latent heat of SAT		257 J/g	Rogerson *et. al.*AIChE Journal, 2003.
Heated mass, *m*		5 g	experimental

Supplementary Table 1 | Material and geometric properties used in the experiment for controllability of local amending.

Supplementary Note1. Phase transition of EVO gel by heat and chemical perturbation.

Sodium Acetate collectively transits with 3 water molecules and has a great stability in supersaturated state appropriate. In case that a gel is swollen with the water manipulator, it effectively swells into the polymer strand retaining supersaturated state depicted in **Supplementary Figure 1a.** Strands and spheres represent polymer chains and water manipulators, respectively. When a perturbation is introduced, manipulators near the polymer chain are detached from the chain and trapped in crystal by entropic pressure that counteracts the attractive interaction with the chain as shown in **Supplementary Figure 1b**. As a result, the polymer chains aggregate and a crystal shield develops as depicted in **Supplementary Figure 1b**.

A chemical perturbation is a way to make supersaturated solution to undergo phase transition by giving energy to overcome the energy barrier ($\Delta G_{\downarrow}r$) . The phase transition may be evoked in many ways such as a touch of seed crystal[11], cavitation effect[12], laser radiation[13] and so on. For simplicity in our research, we touched a seed crystal (sodium acetate tri-hydrate) whose radius exceeds the critical radius.

Furthermore, in a mechanistic viewpoint as shown in **Supplementary Figure 2**, when phase transition to I-mode EVO gel, water manipulators start to crystallize, and polymer strands aggregate. Reversely, heating the gel at 60ºC will alter the gel from V-mode to I-mode. The crystallized water manipulators dissolves, and polymer strands get swelled and segregate. As a result, switching between two modes is reversible. Moreover, by tuning the amount of SBWM after the phase transition, rigidity in V-mode can be locally designated. This enabled a rod with homogeneous softness in I-mode and dramatically different rigidity in V-mode.

Supplementary Note2. Mechanical and selective behavior of EVO gel depending on the fabrication parameters.

EVO gel shows both elastic and plastic behavior. This can be identified in **Supplementary Figure 10**. For the curve drawn by c0v0.25, the curve after waiting for ten seconds keeps falling until going back to 0 compressed length. This behavior means that the sample exerts force till the original state representing elastic behavior. On the other hand, for the curve drawn by c1v0.1, the curve after waiting goes to zero force when compressed length is only about 0.4mm. This behavior means that the sample shows elastic behavior between compressed length 0.5mm to 0.4mm and plastic behavior after which is appropriate for mimicking bone. For the curve drawn by c0.2v0.18 shows intermediate behavior between elastic and plastic behavior which is appropriate for mimicking cartilage. The EVO gel's composition was carefully chosen considering both mechanical behavior and absolute modulus before and after transition.

A rod with one linkage was fabricated to verify the controlled deformation representing the selectivity. The weights with 100g and 1200g were hung to the rod. The rigid part (7 cm) could bare the weight while the soft part (5 cm) could not as shown in Figure 1b. (exactly same as that we designed the gel as shown in Supplementary Figure 16)

Supplementary Note3. Stastiscal analysis of data

R square value represents the coefficient of determination in **Figure 3d** and **3e**. The coefficient of determination is calculated as below.

$$\bar{\mathbf{y}} = \frac{1}{n}\sum_{i=1}^{n} y_i \qquad \text{(S1)}$$

$$SS_{res} = \sum_i (y_i - f_i)^2 = \sum_i e_i^2 \qquad \text{(S2)}$$

$$S_{tot} = \sum_i (y_i - \bar{y})^2 \qquad \text{(S3)}$$

$$\mathbf{R^2 = 1 - \frac{SS_{res}}{SS_{tot}}} \qquad \text{(S4)}$$

where $\bar{\mathbf{y}}$ is the mean of the observed data, SS_{tot} is the total sum of squares, and SS_{res} is the residual sum of squares. The $\mathbf{q^*}$ and γ in **Figure 3d** and **3e** are the values that make the coefficient of determination maximum for each case. The coefficient is determined with the average experimental repetition of 3.84.

Supplementary note4. Scaling of strongly bounded water manipulator

In order to count the number of strongly bounded water manipulator, we starts applying the Poisson-Boltzmann equation to gel single cell as depicted in **Supplementary Figure 7a**.

$$\nabla^2\psi = -\frac{\rho}{\varepsilon_r\varepsilon_0} \quad \text{(S5)}$$

where ψ is electrical potential, $\rho = 2ec_0 \sinh\left(\frac{e\psi}{k_BT}\right)$ is local charge density following the Boltzmannstatics, c_0 is bulk ion concentration, k_B is Boltzmann constant, *T* is absolute temperature, *e* is elementary charge, and $\varepsilon_r\varepsilon_0$ is electrical permittivity. Since 1D polymer strand closes the 2D surface, a unit cell of gel should be treated as 2D geometry. Also amorphorous nature of gel allows us to assume the the circumferential symmetry (**Supplementary Figure 7b**). It simplify the **Equation S5** with linearizing[1] an exponential of charge density, so calld Debye-Huckel approximation.

$$\frac{\mathrm{d}^2\psi}{\mathrm{d}r^2} = -\frac{2ec_0}{\varepsilon_r\varepsilon_0}\frac{e\psi}{k_BT} \quad \text{(S6)}$$

Appropriate boundary condition for $r = 0, \infty$ yields the solution of **Equation S6**.

$$\psi(r) = \psi_0 \exp\left(-\frac{r}{r_d}\right) \quad \text{(S7)}$$

where r_d is Debye length and ψ_0 is zeta potential of gel.

$$r_d = \sqrt{\frac{\varepsilon_r\varepsilon_0 k_BT}{2e^2c_0}} \quad \text{(S8)}$$

For considering effectively bounded water manipulator, we should count excess amount of water manipulator.

$$n^{+}(r) = n(\mathbf{r}) - c_0 = \frac{ec_0}{k_B T}\psi_0 \exp\left(-\frac{r}{r_d}\right) \qquad \text{(S9)}$$

Strongly bounded water manipulator is evaluated by integration of excess amount from 0 to cell diameter and circumferential integration.

$$\pi\xi\bar{c}\bar{\xi} = \frac{1}{2}\pi\xi\int_0^{\xi} n^{+}(r)\mathrm{d}r \qquad \text{(S10)}$$

Upper bar represents the averaged operation and the dimensionless number, potential, and Debye length are expressed as follows.

$$\frac{\bar{c}}{c_0}\frac{\bar{\xi}}{\xi} = \frac{1}{2}\frac{e\psi_0}{k_B T}\frac{r_d}{\xi}\left[1 - \exp\left(-\frac{\xi}{r_d}\right)\right] = \psi_0^{*} l\left[1 - \exp\left(-\frac{1}{l}\right)\right] \qquad \text{(S11)}$$

$$\psi_0^{*} = \frac{1}{2}\frac{e\psi_0}{k_B T}, \qquad l = \frac{r_d}{\xi} \qquad \text{(S12)}$$

For scaling by polymer parameters, The bulk concentration c_0 should be given by polymer volume fraction v_2.

$$c_0 = \frac{\rho_1 V_1}{V_1 + V_2} = \rho_1(1 - v_2) \qquad \text{(S13)}$$

where ρ_1 is number density of water manipulator. Therefore, Debye length and diameter of cell[2] are scaled as follows.

$$r_d = \sqrt{\frac{\varepsilon_r \varepsilon_0 k_B T}{2e^2 \rho_1}}(1-v_2)^{-\frac{1}{2}} \sim O(10^{-11})\,\mathbf{m} \times (1-v_2)^{-\frac{1}{2}} \tag{S14}$$

$$\xi = r_0 v_2^{-\frac{1}{3}} \sim O(10^{-8})\,\mathrm{m} \times v_2^{-\frac{1}{3}} \tag{S15}$$

$$l \sim O(10^{-3}) \times v_2^{\frac{1}{3}}(1-v_2)^{-\frac{1}{2}} < O(10^{-2}) \tag{S16}$$

In this scale, the exponential in **Equation S11** is effectively vanished.

$$\frac{\bar{c}}{c_0}\frac{\bar{\xi}}{\xi} \sim \psi_0^{*} v_2^{\frac{1}{3}}(1-v_2)^{-\frac{1}{2}} \tag{S17}$$

Note that the asymptote of strongly bounded water goes to zero when $v_2 \to 0$ and ψ_0^{*} with $v_2 \to 1$ since exponential in Equation S7 is no longer omitted.Finnally, approximation of last factor in $0 < v_2 < 1$ is applied.

$$\frac{\bar{c}}{c_0}\frac{\bar{\xi}}{\xi} \sim \psi_0^{*}\left(v_2^{\frac{1}{3}} + \frac{v_2^{\frac{4}{3}}}{2}\right) \tag{S18}$$

Consequantly, strongly bounded water manipulator monotonically increses with polymer zeta potential and polymer volume fraction.

Supplementary note5.1. Free energy concerning the first order phase transition

The phase transition involving latent heat is refered as the first order transition. Landau[3] phenomenologically proposed that the free energy is able to be expanded in a power series of the order parameter *m* with the spirit of the mean field theory. The symmetric group generates the set of the order parameter which is finite on the ordered state and zero on the disordered state. In general, The 6th order even polynomial of the order parameter describes the first order transition.

$$F_c - F_{c0} = \alpha(T - T_c)m^2 + \beta m^4 + \gamma m^6 \tag{S19}$$

where $F_c - F_{c0}$ is the crystallization free energy change to reference state, Greeks are some constant, $\alpha > 0, \beta < 0,$ **and** $\gamma > 0$, $T - T_c$ is temperature difference with the critical temperature. We chose the order parameter of the system as fraction of solid component.

$$m \equiv 1 - \frac{n}{n_i} \tag{S20}$$

n is the number of liquid component and n_i is total number of SAT group. The functional form of the order parameter was intentionally set with liquid component, n to extract the bound water expression.Note that, with such definition, we cannot deal with the behavior of liquid state above the critical temperature (maximum of the order parameter is restricted to n_i). However, we are safe from this danger since the transition of supercooling phenomenon would occur below the critical temperature.

For the moment, we should utilize 3 conditions to evaluate the coefficients: 1) latent heat, 2) the discontinuous jump of an order parameter and 3) susceptibility (specific heat) during a transition. Unfortunately, in order to take the discontinuous jump of susceptibility, we have to regard free energy above the critical temperature ($T \rightarrow T_c^+$), which is not defining the order parameter. Therefore, one

needs to reduce the order of the free energy or seek another condition. Near the onset of solidification (positive limit of disordered state i.e. $m \to 0^+$), rigorous consideration of the free energy[4] had been made that a small bump exists. Since we are going to investigate the behavior of an ordered state, a small bump of disordered state can be properly ignored. While this approximation allows a reduction of the number of coefficients, we inevitably lost an opportunity to analyze the order parameter adjacent the critical temperature in a detailed manner. (Note that we cannot produce T^*, T^{**} variables.) However, the equilibrium order parameter describing an ordered state is only factor we should consider. The remaining conditions, the latent heat and a jump of the order parameter constrains the free energy as follow.

$$\left.\frac{\partial F_c}{\partial m}\right|_{m=0} = \left.\frac{\partial F_c}{\partial m}\right|_{m=1} = 0 \qquad \text{(S21)}$$

$$Q^* n_i = T_c \Delta S = T_c \left(\left.\frac{\partial F_c}{\partial T}\right|_{m=0} - \left.\frac{\partial F_c}{\partial T}\right|_{m=1} \right) \qquad \text{(S22)}$$

Equation S21 seems to be more powerful constraints than original condition, but it is natural since the order parameter was defined the number fraction of liquid component which is not sensitive to temperature under single phase state. And Q^* is latent heat per SAT molecules. Thus, the free energy regarding the transition of supercooling of SAT can be read as follow.

$$F_c - F_{c0} = n_i Q^* \left(1 - \frac{T_c}{T}\right)\left(1 - \frac{n}{n_i}\right)^2 \left[\left(1 - \frac{n}{n_i}\right)^2 - 2\right] \qquad \text{(S23)}$$

Supplementary note5.2. Hydrogel induced molecular field consideration

Free energy contribution of a hydrogel in a SAT solution is analogous to Flory's work[5].

$$F_f - F_{f0} = nkT\left[\log[(1-v]_{2r}) + \frac{q^*}{kT}v_{2r}\right] \quad \text{(S24)}$$

The first term of **Equation S24** is considering the mixing phenomenon and the second stands for the polymer-solvent interaction where q^* is the effective interaction energy between polymer strand, analogous to Flory-Huggins parameter. Polymer volume fraction, v_{2r} should be carefully dealt with that **Equation S25** can be only applied to solvent-polymer system, i.e. v_{2r} is function of *n*.

$$v_{2r} = \frac{xn_2}{n + xn_2} \quad \text{(S25)}$$

where n_2 is number of polymer monomer, and *x* is the ratio of the molar volumes of the polymer and solvent. Note that the crystallization free energy, **Equation S24** is applied to red region in **Figure 3b** and molecular field is working in the blue, thus, the control volume for **Equation S24** shrinks with development of the crystal, therefore, the residual SBWM is being *n* itself not nv_{2r} ,

Supplementary note5.3. Evaluation of the proportion of SBWM

The overall free energy of the system can be constituted by addition of **Equation S23** and **S24** as shown in Equation 3 of the manuscript. Differentiating it with respect to *n* yields the chemical potential of the system. Let $c = {}^{n}/_{n_i}, c_2 = {}^{n_2}/_{n_i}$,

$$\mu = -4Q^{*}\left(1-\frac{T}{T_c}\right)(1-c)[(1-c)^2-1] + kT\left[\log\left(\frac{c}{c+xN_2}\right)+\frac{xc_2}{c+xc_2}+\frac{q^{*}}{kT}\left(\frac{xc_2}{c+xc_2}\right)^2\right] \quad \text{(S26)}$$

The proportion of residual bound water we wish to evaluate is c_r such that the chemical potential is set to be equilibrium. q^{*} was evaluated by iteration method using **Equation S26** and transition enthalpy data in the **Figure 3d** since the c_r is function of q^{*} and transition enthalpy relates to both.

Supplementary note6. Diffusive polymerization and Analysis of the Gradient Structure into the EVO gel

Diffusive polymerization consists of three steps that are the diffusing step, the halfway polymerization step, and the complete polymerization step. In the diffusing step, the solution is swelled into the halfway polymerized hydrogel for 30 minutes in room temperature for monolithic integration between two different hydrogels. In the halfway polymerization step, the gel is polymerized in the 60ºC oven for 60 minutes considering the complete polymerization step takes about 90 minutes. This step allows the gel to have a gradient structure which will be discussed in the next paragraph. In the complete polymerization step, the gel is polymerizaed in the 60ºC oven for 90 minutes allowing the gel to be prepared thoroughly.

To verify that the gradient structure is successfully implemented to the EVO gel, we calculated the characteristic diffusion length.

$$L = 2\sqrt{\mathbf{D}t} \qquad \text{(S27)}$$

With diffusion coefficient, $\mathbf{D} = \frac{5 * 10^{-6} cm^2}{s}$ [6-8]and time for 1800 seconds. The calculated diffusion length is about 2mm. According to the fact that we poured the precursor solution before the gel was fully fabricated, this value guarantees that the gradient structure is succesfully implemented.

Supplementary note 7. Measurement of elastic modulus and latent heat

We set up the indentating profile with 3 steps. First, the sample is compressed with the speed of 50μ m/s for 10 seconds. Then, waiting time is set to be 10 seconds to distinguish the sample's elastic and plastic modulus. Finally, the indentation tip returns to the starting position, measuring the elastic force of the sample. The elastic modulus for each sample is determined by the equation below[9]

$$E = \frac{\sqrt{\frac{\pi}{A}}}{2}\frac{dp}{dh} \tag{S28}$$

where, A is truncated area of the sphere-shape tip, $\frac{\mathbf{dp}}{\mathbf{dh}}$ is the slope of the graph when returning to the original position. All indentation curves for control parameters are available in **Supplementary Figure 9.**

Supplementary note8. Local Transformation – Re-programming Mode, Amending State

EVO gel has elasticity in I-mode. This means that one can deform to any shape. After one deforms the shape of the gel, one can fix that shape by giving chemical perturbation to the gel. Taking advantage of this feature, one can intentionally make a certain part of the V-mode gel to I-mode, deform to any shape, and fix the shape which is so called re-programming mode. In this research, by heating only a certain part of the gel, one can change the V-mode gel to re-programming mode and proceed as explained.

We conducted the experiment to investigate transient behavior of EVO gel when local heating. Bone EVO gels (c1v0.11 condition, covered by glass cylinder and sealed with Ecoflex cap) in V-mode were immersed in temperature-controlled water bath (magnetic stirred with 70 RPM) in order to constrain thermal condition. **Supplementary Figure 21a, b**show the temporal change (initial, half converted, and fully converted) of V-mode to I-mode when locally heated (60, 65, 70, 80 ˚C). Higher temperature makes EVO gel transit faster and allows longer time for amending process.

Converted area was exactly equal to heated region (mode interface is exactly same as surface of water bath in each temperature) because conductive heat transfer in gel is effectively smaller than latent heat. Scaling comparison between these two terms can be read as followed.

$$q_{\mathrm{cond}} \sim k\frac{\Delta T}{L}A\Delta t \sim O(10^{-1})\frac{O(10)}{O(10^{-2})}O(10^{-4})O(10^{2})\,J \sim O(1)\,\mathrm{J} \qquad \text{(S29)}$$

$$q_{\mathrm{latent}} \sim mL \sim O(1)O(10^{2}) \sim O(10^{2})\mathrm{J} \qquad \text{(S30)}$$

We presented material and geometric properties in**Supplementary Table 1**. Furthermore, we examined equilibrium state in 80 ˚C local heating as shown in **Supplementary Figure 21c**. After transformation of the part under surface of water bath is completed, upper part starts to convert by conductive heat transfer as expected by equation S29, S30. Therefore, we can safely conclude that local amending process is controllable.

Supplementary note9. DOF Addition mechanisms and procedure

For DOF addition, the same mold with the kinetic bar (**Supplementary Figure 16**) but with different length of each part is used.Individual part of bone and cartilage is 19 mm each. The precursors used for bone and cartilage ars the same with other experiments which are c0.1v0.11 and c0v0.25, respectively.

DOF addition process consists of two big steps. First step is diffusion stage. Two different gels in I-mode contact each other and let them diffuse to each other. As the gel is in I-mode, polymer chains and water manipulaters can easily move to each other. This process is depicted in **Supplementary Figure 18**. After the diffusion step, a chemical perturbation is given to the gels. This causes the crystallization to propagate over the interface of two different gels. In the process of propagation, a synergetic effect occurs making two gels attach to each other almost completely. The synergetic effect includes three parts. First one is the crystal shielding effect around the interface. As crystal shield is newly created when crystal propagates around the bulky part and the interface part at the same time, the newly made shield spontaneously affix two different gels. Second one is the interlocking effect through the interface. Though two gels diffuse to each other, they must have interface between them due to chemical cross-linking nature. However, in the process of diffusion, this interface invade into themselves so when the crystallization is complete, each gel’s side facing the interface is interlocked by each side. Third one is enhanced diffusion of polymer chains by crystallization propagation. As solid crystal is created through the propagation process, the polymer chains aggregate each other accelerating the diffusion.

Supplementary note10. Advantage of volumetric dehydration in EVO

Although polymer aggregation during dehydration contributes to enhancement of mechanical modulus in both cases, the main differences between evaporation and EVO are the domain of dehydration and net outward water flux as depicted in **Supplementary Figure 19**. In evaporation, outward water flux develops in surface of gel while EVO can dehydrate a gel volumetrically and keep water component. We explored micro dehydration phenomenon near single polymer cell in the manuscript (**Figure 3b**). The result was extensively expanded to entire system and well matched to the experiment (**Figure 3e**), which suggests that phase transition of water manipulator dehydrates an entire volume of EVO gel. Furthermore, reversibility of EVO presented in **Figure 2c** of the manuscript shows absence of outward water flux.

Volumetric dehydration by phase transition of water manipulator has two advantageous properties in altering mechanical modulus compared to evaporation; speed (domain of dehydration) and reversibility (no outer water flux) of the transition.

Supplementary note11. Mechanistic analogy between EVO and Salting-out

Sodium acetate interacts more strongly with water molecules in a solid phase than in a liquid state, leading to its negative and large latent heat of solidification. Therefore, the component that mainly interacts with water molecules is different according to the mode of EVO gel as shown in **Supplementary Figure 20a**.

In I-mode, three components of EVO gel (polymer, salt, and water) almost equally interact similar to "Salting-in" state. The solubility of polymer should be high which leads to a stable gel state is achieved. However, when the EVO gel undergoes phase transition, a set of sodium acetate ion vigorously attracts three water molecules to build energetically stable tri-hydrate crystal. In this process, polymer strands become de-swollen and aggregate which is very similar to precipitation of polymer in "Salting-out". Mechanistic analogy between EVO and salting-out is depicted in **Supplementary Figure 20b**. Note that, in salting-out, the polymer is precipitated with continuously controlled salt concentration while EVO performs the process discontinuously by phase transition of salt hydrates. Gel solubility of V-mode in **Supplementary Figure 20b**is qualitatively estimated based on chemical potential of water manipulators with constant polymer volume fraction.

The salting-out phenomenon would play a crucial role in discontinuous formation of salt hydrate crystal inducing the aggregation of polymer strand that enhances the mechanical property of EVO gel. It is expected that this effect might contribute to exponent of Equation 4 in the manuscript by ~2.3.

Supplementary References

1 Hückel, E. & Debye, P. The theory of electrolytes: I. lowering of freezing point and related phenomena. *Phys. Z* **24**, 1 (1923).

2 Saraydın, D., Karadag, E., Işıkver, Y., Şahiner, N. & Güven, O. The influence of preparation methods on the swelling and network properties of acrylamide hydrogels with crosslinkers. *Journal of Macromolecular Science, Part A* **41**, 419-431 (2004).

3 Landau, L. The theory of phase transitions. *Nature* **138**, 840-841 (1936).

4 Jin, X., Medina, M. A., Zhang, X. & Zhang, S. Phase-change characteristic analysis of partially melted sodium acetate trihydrate using DSC. *International Journal of Thermophysics* **35**, 45-52 (2014).

5 Flory, P. J. & Rehner Jr, J. Statistical mechanics of cross-linked polymer networks I. Rubberlike elasticity. *The journal of chemical physics* **11**, 512-520 (1943).

6 Aktaş, D. K., Evingür, G. A. & Pekcan, Ö. Study on swelling of hydrogels (PAAm) at various temperatures by using fluorescence technique. *Journal of materials science* **42**, 8481-8488 (2007).

7 Mironova, T. & Kraiski, A. Determination of Diffusion Coefficient in Hydrogel. *KnE Energy*, 429–436 (2018).

8 Pavesi, L. & Rigamonti, A. Diffusion constants in polyacrylamide gels. *Physical Review E* **51**, 3318 (1995).

9 Hoffman, D. Measuring the elastic modulus of polymers using the atomic force microscope. (2010).

10 Sennakesavan, G., Mostakhdemin, M., Dkhar, L., Seyfoddin, A. & Fatihhi, S. Acrylic acid/acrylamide based hydrogels and its properties-A review. *Polymer Degradation and Stability*, 109308 (2020).

11 Rogerson, M. A., & Cardoso, S. S. Solidification in heat packs: I. Nucleation rate. AIChE journal **49(2)**, 505-515 (2003).

12 Rogerson, M. A., & Cardoso, S. S. Solidification in heat packs: II. Role of cavitation. AIChE journal **49(2)**, 516-521 (2003).

13 Hua, T., Valentín-Valentín, C., Gowayed, O., Lee, S., Garetz, B. A., & Hartman, R. L. Microfluidic laser-induced nucleation of supersaturated aqueous glycine solutions. Crystal Growth & Design **20(10)**, 6502-6509 (2020).

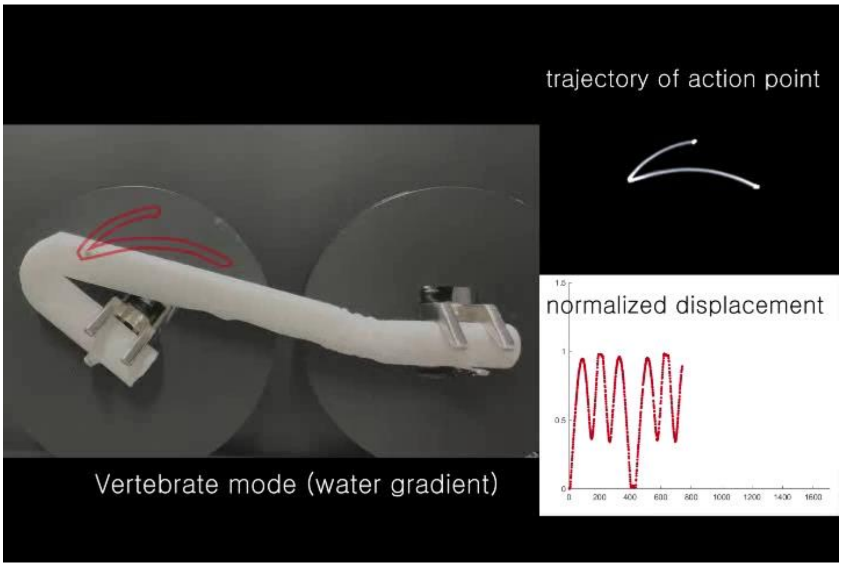

Supplementary Movie 1. Kinematics of I-mode and V-mode

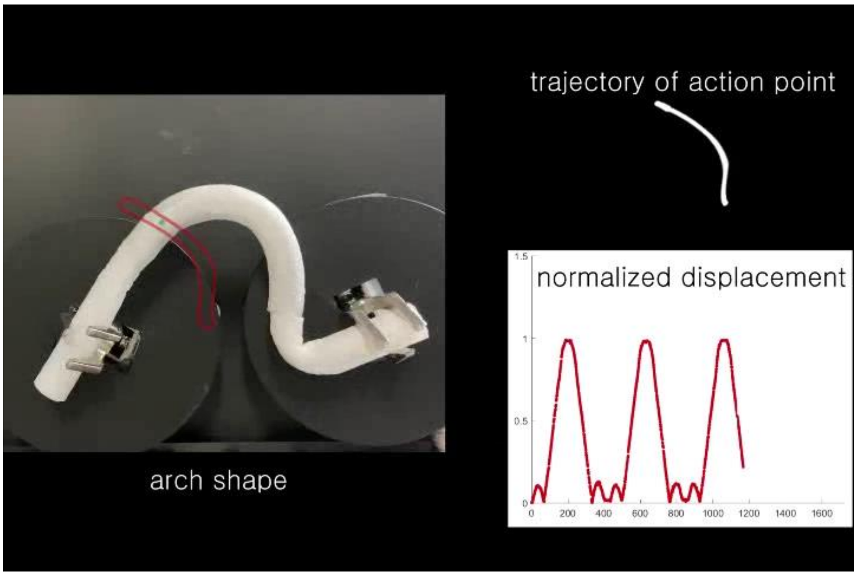

Supplementary Movie 2. Kinematics of re-programmed V-mode
